# Emerging role of immunogenic cell death in cancer immunotherapy

**DOI:** 10.3389/fimmu.2024.1390263

**Published:** 2024-05-10

**Authors:** Kei-ichiro Arimoto, Sayuri Miyauchi, Mengdan Liu, Dong-Er Zhang

**Affiliations:** ^1^ Moores Cancer Center, University of California San Diego, La Jolla, CA, United States; ^2^ School of Biological Sciences, University of California San Diego, La Jolla, CA, United States; ^3^ Department of Pathology, University of California San Diego, La Jolla, CA, United States

**Keywords:** immunogenic cell death (ICD), anti-tumor immune response, USP18 (UBP43), PLK2, immunotherapies, tumor micreoenvironment (TME), tumor biology, interferon

## Abstract

Cancer immunotherapy, such as immune checkpoint blockade (ICB), has emerged as a groundbreaking approach for effective cancer treatment. Despite its considerable potential, clinical studies have indicated that the current response rate to cancer immunotherapy is suboptimal, primarily attributed to low immunogenicity in certain types of malignant tumors. Immunogenic cell death (ICD) represents a form of regulated cell death (RCD) capable of enhancing tumor immunogenicity and activating tumor-specific innate and adaptive immune responses in immunocompetent hosts. Therefore, gaining a deeper understanding of ICD and its evolution is crucial for developing more effective cancer therapeutic strategies. This review focuses exclusively on both historical and recent discoveries related to ICD modes and their mechanistic insights, particularly within the context of cancer immunotherapy. Our recent findings are also highlighted, revealing a mode of ICD induction facilitated by atypical interferon (IFN)-stimulated genes (ISGs), including polo-like kinase 2 (*PLK2*), during hyperactive type I IFN signaling. The review concludes by discussing the therapeutic potential of ICD, with special attention to its relevance in both preclinical and clinical settings within the field of cancer immunotherapy.

## Introduction

To improve the efficacy of anti-cancer immunotherapies, numerous studies have concentrated on immune cells, exploring strategies to target the immunosuppressive tumor microenvironment (TME) such as T-cell suppressors and inhibitory immune checkpoints (1–4). Indeed, some cancer patients derive clear benefits from these T-cell-based immunotherapies (5, 6). However, unfortunately, most solid tumors are “cold” tumors with low immunogenicity (the ability of tumor antigen to trigger immune response), limited immune cell infiltration, and insufficient immunological responses. This results in poor responses by approaches that solely enhance T-cell function (7). Consequently, many oncologists have shifted their focus towards developing methods to transition cold tumors into “hot” tumors in combination with T-cell-based therapy, thereby augmenting intratumor immunogenicity. This process is commonly referred to as ‘inflaming the TME’ (8). To inflame the TME favorably for anti-tumor immunity, various approaches, such as oncolytic viruses, have been proposed (9). Among these, the induction of immunogenic cell death (ICD) in tumors has garnered significant interest (10–12).

## Features of ICD

**Figure 1:**
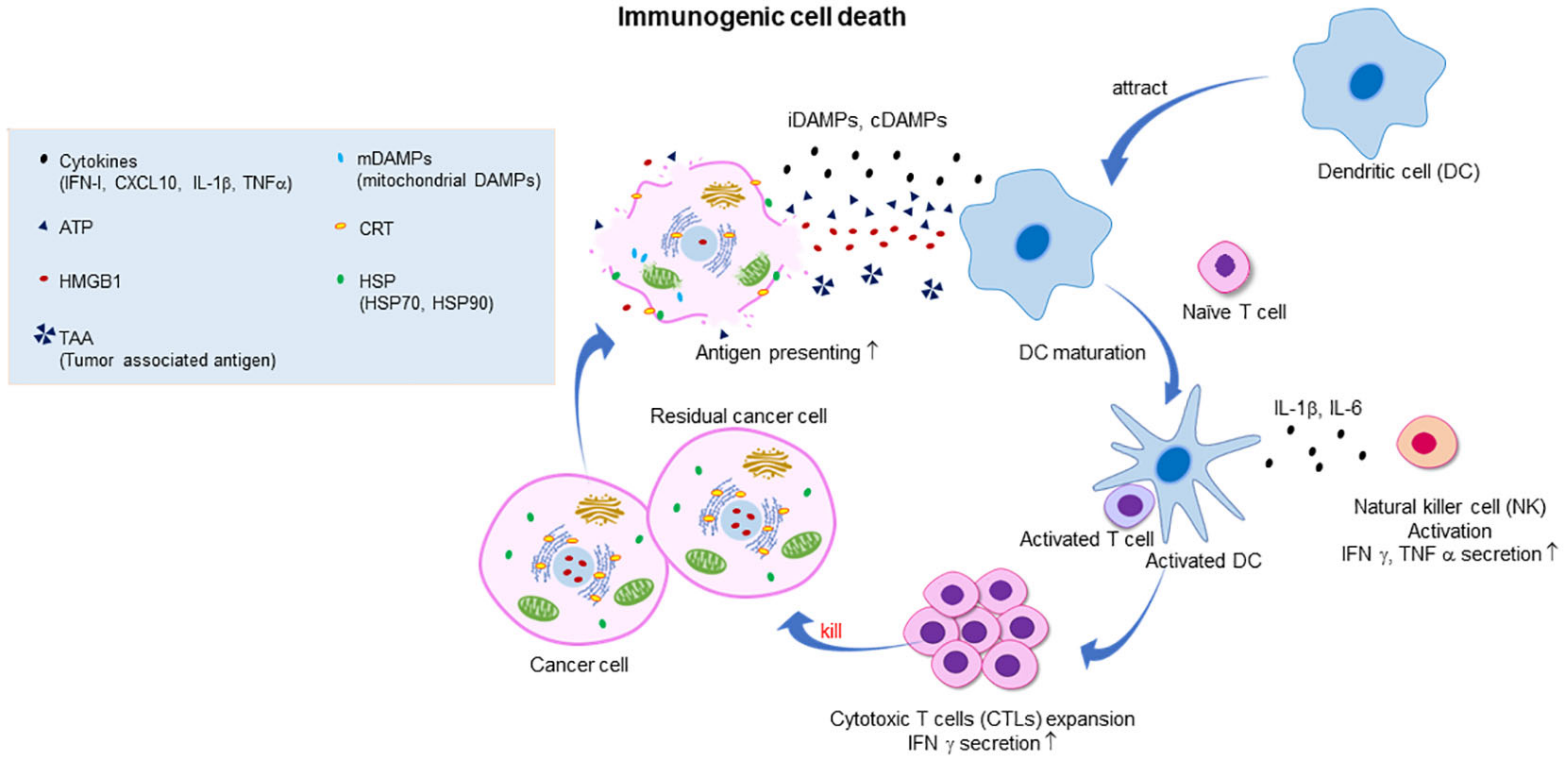
Illustration of the mechanism of cancer immunogenic cell death (ICD) and the subsequent activation of the antitumor immune response. Dying cancer cells express various constitutive damage-associated molecular patterns (cDAMPs) and inducible DAMPs, including the release of high mobility group box 1 (HMGB1) from the nucleus, translocation and cell surface exposure of calreticulin (ecto-CRT) and heat shock proteins HSP70/90, and extracellular secretion of ATP, cytokines (such as type I IFN), chemokines (such as CXCL10), and nucleic acids. Exposure to DAMPs serves as a “find me” signal which recruits immature dendritic cells (DC) to tumor microenvironment (TME) and induces the maturation of DC. Ecto-CRT provides a pro-phagocytic “eat me” signal that promotes the phagocytosis of antigens by DC. In addition, HMGB1 and HSP70/90 assist in promoting the processing of phagocytic cargo by binding to toll-like receptors (TLRs), thereby escalating antigen engulfment, processing, and cross-presentation to CD8^+^ T cells to mediate robust tumor-specific immune response and protective immunological memory. Ecto-HSP70 and HSP90 also stimulate NK cell lysis. Primed CTLs elicit direct cytotoxic response and eradicate remaining tumor cells through the generation of IFN-γ, perforin-1 and granzyme B.

ICD, discovered by Guido Kroemer’s group in 2005 (13–15), is a form of cell death that activates the immune system, initiating an inflammatory response and facilitating the recognition of dying cells by the immune system (**Figure 1**). This feature is pivotal in contrast to non-immunogenic cell death, such as canonical apoptosis, where the immune system remains unaffected or may even be suppressed (e.g., by the expression of Transforming Growth Factor-β (TGF-β)). ICD is characterized by the release or cell-surface expression of highly immunostimulatory damage-associated molecular patterns (DAMPs) from dying tumor cells with ruptured cell membranes, followed by the induction of the innate and adaptive tumor-specific immune responses. The extracellular release of the nuclear protein high mobility group box 1 (HMGB1) and adenosine triphosphate (ATP), both termed as ‘alarmins,’ acts as an attractant and activates antigen-presenting cells (APCs), such as dendritic cells (DCs). Extracellular HMGB1 binds to the receptors (e.g., Toll-like receptor 2 (TLR2), TLR4, and RAGE (receptors for advanced glycation end-products)) of immature DCs which cause DC maturation and cytotoxic T lymphocytes (CTLs) activation (16). Extracellular ATP acts as a powerful chemotactic agent for APCs and their precursors by binding to purinergic receptors P2Y2R and P2X7R to promote interleukin 1β (IL-1β) and IFN-γ expression and CD8^+^ T-cell priming (17, 18). Simultaneously, the display of calreticulin (CRT), normally residing inside the endoplasmic reticulum but translocated to the dying cell’s surface, serves as an “eat-me” signal for phagocytes like macrophages (17, 19–22). The immunogenicity of CRT expressing cells could be effectively abolished by CRT inhibition with blocking antibodies, or by CRT knockdown with specific small interfering RNAs (21). Conversely, mRNA expression of CRT in cancer cells affects the composition and density of infiltrating immune cells (23). Indeed, CRT expression is mainly linked to CTLs and DCs infiltration in various types of cancer, such as colorectal, ovarian, and breast cancers. The membrane expression of heat shock proteins, such as HSP70 and HSP90 during ICD, also contributes to immune stimulation (24). Both HSP70 and HSP90 is usually located in the intracellular compartment, however, these HSPs translocate to cell surface during ICD. These ecto-HSP70 and HSP90 can interact with receptors on the surface APCs (e.g., CD40 and CD91) and enhance the immunogenicity of dying cells, results in the cross-presentation of cancer cell antigens to major histocompatibility complex (MHC)-I molecules and subsequent activation of CD8^+^ T-cells (16, 25, 26). Antigens from dying cancer cells are taken up and processed by APCs, which then present these antigens through their MHC-I molecules to T cells (27). Above mentioned DAMPs are called constitutive DAMPs (cDAMPs) and critically important to initiate ICD-mediated anti-tumor immunity (8). In addition to DAMPs, pathogen-associated molecular patterns (PAMPs), including endogenous RNA and double-stranded DNA (dsDNA) (endogenous and exogenous if oncolytic virus-mediated ICD), can also be released during ICD. PAMP sensors are responsible for the release of type I IFNs and C-X-C motif chemokine ligand 10 (CXCL10) through IRF3 and NF-κB pathways (28–31). Absent in melanoma 2 (AIM2) inflammasome can sense dsDNA released by ICD cells, thereby inducing IL-1β secretion (32). These cytokines have been implicated in DC maturation, T, and natural killer (NK) cell recruitment. Type I IFNs enhance the cytotoxicity of CTLs and NK cells and promote the cross-presentation of DCs (33, 34). These cytokines and chemokines are transcriptionally induced therefore called inducible DAMPs (iDAMPs) (8). As such, tumor cells undergoing ICD act as an endogenous vaccine, attracting activated immune cells into the tumor microenvironment or draining lymph node.

Importantly, various tumor-associated neo-antigens derived from cancer-specific somatic mutations or gene fusions should be present during ICD. Tumor-specific peptides displayed by APCs can activate T cells, particularly the activation of CTLs, which are then licensed to engage in tumor-specific immunity. This principle of ICD can be confirmed by a vaccination scenario known as the ‘gold standard vaccination assay,’ where inoculation of mice with tumor cells killed by ICD induction prevents the subsequent growth of live tumor cells (35, 36). Importantly, traditional cancer treatment regimens, such as chemotherapy and radiation, are related to ICD induction. While not all chemotherapeutic agents induce ICD, some, such as anthracyclines (e.g., Doxorubicin and mitoxantrone) and oxaliplatin, are known ICD inducers (18, 37). In human breast and colorectal cancer patients treated with anthracyclines or oxaliplatin, favorable clinical outcomes were found to be associated with an increased number of cytotoxic CD8^+^ T cells within the tumor (38, 39). The loss of DC function was identified as a negative predictor of the therapeutic response to anthracyclines or oxaliplatin in both clinical and preclinical settings (40, 41).

## Evolution of ICD in cancer

**Figure 2:**
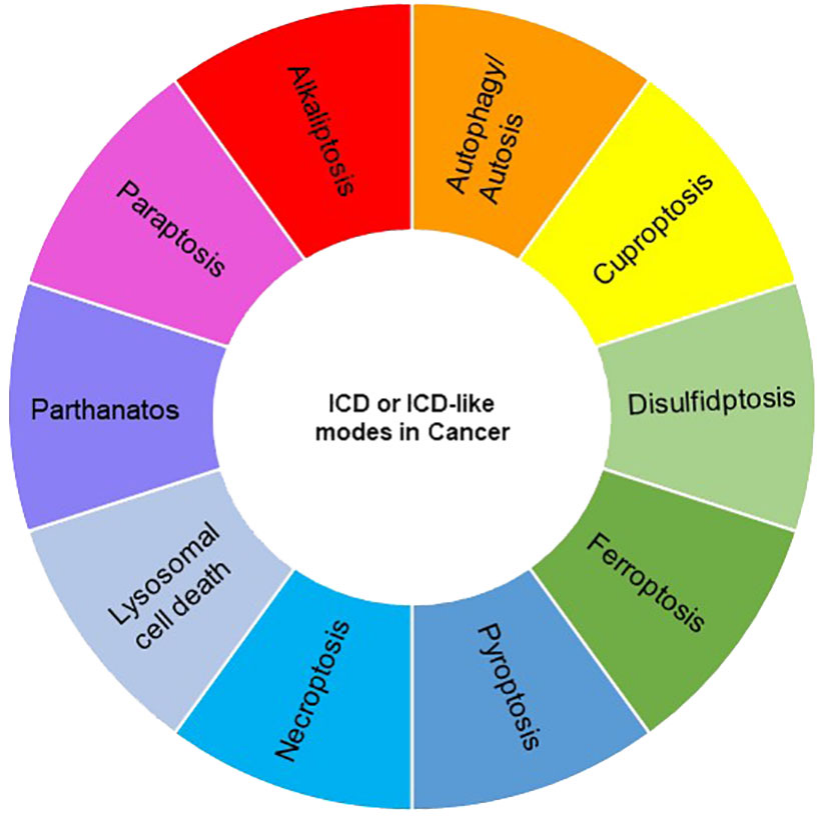
A landscape of currently discovered ICD or ICD-like modes in cancer.

**Figure 3:**
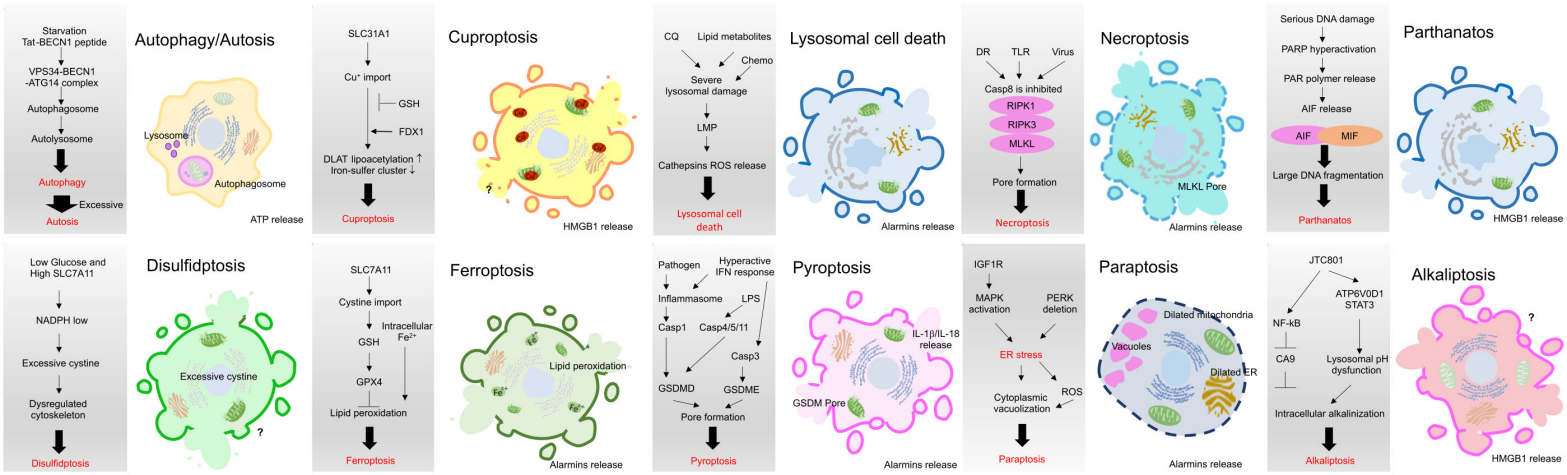
Brief mechanistic description of current ICD or ICD-like modes in cancer. Necroptosis, ferroptosis, and pyroptosis have been extensively studied and are regarded as forms of ICD, despite their distinct mechanisms. Recent studies have started to reveal that other modes may be considered ICD-like if their consequences align with the features of ICD, namely, the release of damage-associated molecular patterns (DAMPs) and the induction of the innate and adaptive immune responses.

Various types of cell death, such as apoptosis, necrosis, alkaliptosis, anoikis, autosis, autophagy, cuproptosis, disulfidptosis, entosis, erebosis, ferroptosis, lysosomal cell death (LCD), methuosis, mitoptosis, necroptosis, NETosis, oxeiptosis, paraptosis, parthanatos, pyroptosis, and immunogenic cell death (ICD), have been identified, exhibiting either caspase-dependent or -independent characteristics. While ICD is classified as one of the cell death modes, other modes may be considered ICD-like if their consequences align with the features of ICD, namely, the release of damage-associated molecular patterns (DAMPs) and the induction of the innate and adaptive immune responses. Notably, necroptosis, ferroptosis, and pyroptosis have been extensively studied and are regarded as forms of ICD, despite their distinct mechanisms (42, 43). In recent years, growing evidence suggests that some other modalities of cancer cell death also exhibit ICD features. In this review, we explore and discuss several modes of cancer cell death with ICD phenotypes, focusing particularly on recent reports within the field of cancer immunotherapy (**Figures 2**, **3**).

### Necroptosis

Cell death is generally classified as accidental cell death (ACD) or regulated cell death (RCD), a genetically/molecularly controlled process. Necrotic cell death, a representative form of ACD, has long been considered a non-programmed and uncontrolled cell death. However, with research in over the past decade, we now understand that morphologically necrotic-like cell death is not only accidental but also programmed, referred to as ‘necroptosis,’ and considered one of the modes of ICD (44). The necroptotic phenotype (controllable necrosis) was first observed in 1996 in cowpox virus infected LLC-PK1 pig kidney cells expressing cytokine response modifier A (CrmA), a viral caspase 1/8 inhibitor (45). In 1998, tumor necrosis factor-α (TNFα) was found to induce necroptosis of L-M cells (a mouse fibroblast cell line) in the presence of a caspase 8 inhibitor, suggesting that caspase 8 negatively regulates necroptosis (46). Today, it is well known that necroptosis can be triggered by TNFα, TNF-related apoptosis-inducing ligand (TRAIL), and Fas ligand, which activate the death receptor-mediated apoptosis pathway and lead to necroptosis when CASP8 activation is prevented by pharmacological caspase inhibitors (e.g., Z-VAD-FMK) or genetic depletion of fas-associated via death domain (FADD) (47, 48). Necroptosis is also triggered by the stimulation of pattern recognition receptors (PRRs) such as Toll-like receptors (TLRs) 3 and 4, DNA-dependent activator of interferon-regulatory factors (DAI), or cyclic GMP-AMP synthase (cGAS) (49–51). Through signal transduction by PRRs, receptor-interacting protein kinase (RIPK) 1 is activated, RIPK3 is recruited, and a necrosome is formed (52, 53). RIPK1/RIPK3 further activates the mixed lineage kinase domain-like pseudokinase (MLKL) (54, 55). MLKL translocates to the plasma and cytoplasmic membranes and promotes cell membrane rupture and cell death, with simultaneous spilling of intracellular content containing pro-inflammatory cytokines and DAMPs (56). The emission of intracellular contents from necroptotic cancer cells, together with the released cytokines and chemokines, renders necroptotic cells immunogenic and thus able to elicit tumor-specific CD8^+^ T cell-mediated responses, resulting in potent anticancer responses (43).

Preventive immunization (gold standard vaccination assay) of mice with necroptotic cancer cells induced by ectopic RIPK3 expression can induce antitumor immunity. In a B16-OVA tumor model and even a RIPK3-deficient CT26 tumor model, MLKL mRNA treatment induced necroptotic cell death and subsequent antitumor immunity (57). Additionally, AAV-induced RIPK3 expression can promote necroptosis in the TME, enhance neoantigen presentation activity, and induce tumor-specific CD8^+^ T-cell priming, resulting in secondary tumor control and improved therapeutic efficacy of immune checkpoint blockers in various tumor mouse models (58). Decreased RIPK3 expression has been reported in several types of cancer patients’ samples (59–62). In head and neck squamous cell carcinoma (HNSCC) patients, the loss of RIPK1 and RIPK3 function caused by promoter hypermethylation is closely related with poor prognoses (63, 64). In hepatocellular carcinoma (HCC) patients, increased RIPK1, RIPK3, and phosphorylated MLKL levels were positively correlated with increases in tumor-infiltrating CD3^+^ and CD8^+^ T-lymphocytes (65). Furthermore, it has been reported that a combination of radiotherapy, chemotherapy, and hyperthermia with Z-VAD-FMK, a necroptosis inducer/pan-caspase inhibitor, can increase macrophage activation, retard tumor growth, and induce immune cell infiltration into tumors through the release of DAMPs in B16 melanoma (66). These reports indicate that necroptosis by the activation of RIPK1/RIPK3/MLKL in the TME can induce APC maturation through the release of DAMPs and promote strong antitumor immunity by inducing tumor-specific CD8^+^ T-cells.

### Ferroptosis

Ferroptosis, initially identified in 2012 by Brent Stockwell’s group, represents an iron-dependent form of non-apoptotic cell death that has recently been attributed with antitumor immune effects (67, 68). Ferroptosis is driven by excessive iron accumulation in the cell, leading to higher lipid reactive oxygen species (ROS) and unrestricted lipid peroxidation. This ultimately results in plasma membrane damage and rupture, leading to the emission of DAMPs. During ferroptosis, the oxidation of antigens and DAMPs, including HMGB1, can alter their antigenic features, thereby enhancing the immunogenicity of ferroptosis (43, 69).

Glutathione (GSH) and GSH biosynthesis pathway proteins such as solute carrier family 7 member 11 (SLC7A11) and glutathione-glutathione peroxidase 4 (GPX4) can convert lipid peroxides to lipid alcohols, which suppress intracellular iron-induced lipid ROS (67). Therefore, conditions that inhibit glutathione biosynthesis or GPX4 can induce ferroptosis. Indeed, specific agents such as erastin (an SLC7A11 inhibitor) and RSL3 (a GPX4 inhibitor) can trigger ferroptosis, selectively eliminating cancer cells (70, 71). Radiotherapy is known to induce ICD and recent evidence suggest that radiation may induce ferroptosis by increasing lipid ROS accumulation (72, 73). Indeed, the depletion of ferroptosis-related gene reduces the efficacy of radiotherapy (72). Crucially, in preclinical models, a vaccination of ferroptotic cells protected against re-challenge with fibrosarcoma, providing critical evidence of ferroptosis as a form of ICD. A recent, important finding revealed a strong increase in lipid peroxidation in the early phase of ferroptosis, but not in the late phase. Early or late ferroptotic MCA205 cells were assessed using the ‘gold standard vaccination assay’ in immunocompetent C57BL/6J mice. Interestingly, re-challenging “vaccinated” mice with live MCA205 cells revealed that only early ferroptotic cancer cells could induce a protective immune response against the fibrosarcoma (74). Moreover, it was demonstrated that extracellular HMGB1 and ATP were necessary for ICD caused by early ferroptotic cells (74). Specifically, pharmacological blockade of the ATP receptor (P2X7) using oxiATP reversed the tumor-protective effects of vaccination with early ferroptotic cancer cells. Finally, sorafenib, approved by the FDA in 2006 for late-stage renal cell carcinoma (RCC) management and in 2007 for advanced-stage hepatocellular carcinoma (HCC) therapy, is known to induce ferroptosis in multiple cancer cell lines (75, 76). Sorafenib triggers ferroptosis through either mitochondrial iron accumulation or SLC7A11 inhibition (77). While some HCC patients benefit from sorafenib treatment, drug resistance typically develops. In recent years, researchers have been exploring new approaches to reverse sorafenib resistance in HCC by elucidating the mechanisms of resistance to sorafenib (78).

### Disulfidptosis

It has recently been discovered that abnormal intracellular disulfide accumulation under glucose starvation conditions can induce a previously uncharacterized form of regulated cell death, referred to as ‘disulfidptosis’ (79). Surprisingly, disulfidptosis requires glucose deprivation and high expression of SLC7A11, which has a negative role in ferroptosis induction, as above mentioned. This indicates that SLC7A11 plays an important role in either ferroptosis or disulfidptosis induction, depending on cellular conditions (80). Indeed, despite the well-established pro–cell survival function of SLC7A11 in the context of ferroptosis induction, other studies have uncovered an unexpected role of SLC7A11 in promoting cell death under glucose deprivation conditions (79, 81–83). Several cancer cells rely on SLC7A11 to import cystine to maintain redox balance and cell survival. To regulate this balance, cells utilize the molecule nicotinamide adenine dinucleotide phosphate (NADPH) to rapidly convert toxic disulfides into non-toxic molecules. Under glucose-starvation conditions, NADPH was severely depleted in cells overexpressing SLC7A11, and disulfides such as cystine accumulated abnormally. The excessive accumulation of disulfide leads to the formation of abnormal disulfide bonds between actin backbone proteins, causing the disintegration of the cytoskeletal protein network and subsequent cell death (79). Thus, disulfidptosis represents a unique mode of cell death distinct from other metal-induced cell death forms, such as cuproptosis and ferroptosis.

As the study of disulfidptosis is in its infancy, we cannot conclusively state that disulfidptosis has ICD potential. However, gene expression analysis during disulfidptosis shows a correlation with the activation of gene sets for immune cell infiltration in the TME, which is one important result of ICD (84–87). Furthermore, it has been recently reported that a GLUT1 inhibitor (known to induce disulfidoptosis) can induce ICD in HCC (88). Further studies are needed to investigate the role of disulfidptosis in cancer immunotherapy.

### Cuproptosis

Copper (Cu) is an essential element in virtually all living organisms, and consequently, low levels of Cu in cells are harmful. Conversely, excessive intracellular copper accumulation also triggers cell death. Recently, a novel form of Copper (Cu)-dependent programmed cell death, termed ‘cuproptosis,’ was reported by Tsvetkov et al. in 2022, suggesting a new strategy for cancer therapeutics (89, 90). The main morphological features of cuproptosis include mitochondrial shrinkage, endoplasmic reticulum injury, and plasma membrane rupture, which is crucial for its ICD potential. The exact molecular mechanism underlying cuproptosis remains unclear, but recent studies have proposed some potential mechanisms (89, 90). The mitochondrion is a major target of Cu-induced cell death. Intracellular Cu targets and binds to lipoylated components in the tricarboxylic acid (TCA) cycle, and aggregation of these Cu-bound lipoylated mitochondrial proteins, such as dihydrolipoamide S-acetyltransferase (DLAT), a subunit of the pyruvate dehydrogenase complex, and the subsequent reduction in iron-sulfur (Fe–S) clusters, facilitates proteotoxic stress and induces cuproptosis. Cu ionophores, such as elesclomol, bind extracellular Cu and transport it to intracellular compartments, inducing cuproptosis. A genome-wide CRISPR screen identified mitochondrial ferredoxin 1 (FDX1) and lipoyl synthase (LIAS) as key regulators of cuproptosis, and genetic knockout of either FDX1 or LIAS attenuates Cu ionophore-induced cell death (89, 91). FDX1 is known to contribute to both DLAT lipoylation and Fe-S cluster proteins degradation. Importantly, Cu chelators, such as tetrathiomolybdate (TTM), inhibit cuproptosis, whereas inhibitors of ferroptosis (Ferrostatin-1), necroptosis (Necrostatin-1), and oxidative stress (N-acetylcysteine; NAC) failed to suppress elesclomol-induced cuproptosis, suggesting that cuproptosis is mechanistically distinct from other forms of cell death (89, 92, 93). Copper complexes containing polypyridine ligands have been reported to enter the endoplasmic reticulum (ER) *in situ*, leading to increased ROS levels and ER-stress-induced ICD in breast cancer cells (94). Recent study has revealed that the combination of elesclomol and Cucl2 combination induces cuproptosis in non-small-cell lung cancer (NSCLC) cells and leads to the HMGB1 release (95). Interestingly, elesclomol selectively induces cuproptosis in melanoma and leukemic cells, suggesting its potential use in clinic (96). Furthermore, several anti-cancer effects have been evaluated using copper-based nanomaterials (97). The discovery of cuproptosis has prompted many researchers to explore its potential use in hepatocellular carcinoma therapy as hepatic cells are particularly rich in mitochondria compared to the other cell types, indicating a distinct vulnerability to cuproptosis in HCC (98). Conversely, several studies have found significantly upregulated Cu levels in hepatic tumor tissue and serum of HCC cancer patients, with elevated Cu levels in tumor cells contributing to immune escape by enhancing PD-L1 expression (99, 100). In this context, several cuproptosis-related genes are currently under investigation to understand elesclomol resistance and provide insight into the potential use of cuproptosis induction for HCC therapy (101–103).

### Paraptosis

Paraptosis, first reported by Bredesen’s group in 2000, is characterized by the extensive vacuolization of ER components and mitochondria swelling, while lacking apoptotic features. Paraptosis is a type of caspase-independent programmed cell death (104). In most cases, paraptosis is mediated by human insulin-like growth factor I receptor (IGFIR) and induced by the downstream MAPK/ERK and JNK/SAPK pathways following ER stress pathway activation. Therefore, paraptosis can be rescued by specific inhibition of these kinases. Indeed, the MAPK activator elaiophylin induces paraptosis in ovarian cancer cells, and gene expression analysis showed that elaiophylin activates the ER-stress pathway, suggesting that ER-stress pathway activation is crucial for the execution of paraptosis (105). A member of TNF receptor superfamily, TNFRSF19, promotes ER-stress via MAPK pathway activation and induces paraptosis in triple negative breast cancer cells (106). A recent study demonstrated that the deletion of the pancreatic ER kinase (PKR)-like ER kinase (PERK) in cancer cells induces paraptosis (107). During ER stress, autophosphorylated PERK dissociates from its negative regulator and phosphorylates several targets, including the eukaryotic translation initiation factor 2α (eIF2α), which triggers the expression of the activating transcription factor 4 (ATF4) that transiently contributes to cancer cell survival. Paraptosis is triggered by proteostasis alterations, a process heavily dependent on the SEC61 translocon complex. Dysregulation of the SEC61 complex by the regulatory subunit SEC61β drives paraptosis by altering the trafficking of proteins through the ER. PERK and ATF4 serve as upstream negative regulators of SEC61β-mediated paraptosis. However, the mechanistic insights of the PERK, ATF4, and SEC61β crosstalk in the regulation of anti-tumor immunity are yet to be elucidated. Most importantly, PERK inhibition in ER-stressed malignant cells triggers DAMPs release and activates anti-tumor T cell immune responses, suggesting that paraptosis is ICD in cancer cells (107). Interestingly, it has also been reported that paraptosis is triggered by natural products, showing the ICD potential of paraptosis. Morusin, a prenylated flavonoid isolated from the root bark of the Morus mulberry plant, could effectively induce paraptosis-like epithelial ovarian cancer cell death by amplifying the oxidative stress of the ER and mitochondria (108). Another study has found that the natural anthraquinone compound, novel rhein derivative 4a, induces paraptosis-like cell death in ovarian cancer cells (109). Indoleamine 2,3-dioxygenase (IDO), which is upregulated in tumor cells, can reprogram tryptophan/kynurenine metabolism to facilitate immune escape. IDO inhibitor can reverse the tumor microenvironment and elicit the host immune system (110). Therefore, a therapeutic strategy synergizing paraptosis induction and IDO inhibition may be greatly advantageous in activating systemic immunity. Indeed, a paraptosis inducer prepared by the assembly of copper ions (Cu2+), morusin, and IDO inhibitor (NLG919) through noncovalent interactions could induce cancer cell paraptosis through mitochondrial and ER vacuolation. This contributes to releasing large amounts of DAMPs to recruit DCs for activating antitumor immunity in a 4T-1 breast cancer mouse model (111).

### Parthanatos

Parthanatos is a form of RCD characterized by necrotic-like morphology. Poly (ADP-ribose) polymerase-1 (PARP-1), a nuclear protein that plays a crucial role in DNA repair, genomic stability, and transcription is the key molecule in the parthanatos mechanism (112, 113). Activation of PARP-1 by parthanatos induces the release of immunogenic alarmins, primarily of HMGB1. Parthanatos is triggered by the hyperactivation of PARP-1 in response to extremely high and prolonged alkylating DNA damage induced by ROS or alkylating agents, resulting in the depletion of cellular energy, the mitochondrial release of apoptosis-inducing factor (AIF), and the production of excess poly (ADP-ribose) (PAR) polymers, followed by large-scale DNA fragmentation (114). In 2016, macrophage migration inhibitory factor (MIF) nuclease was identified as a crucial factor in the induction of parthanatos by forming a MIF/AIF complex (115). Several anti-cancer drugs efficaciously induce parthanatos-dependent cancer cell death in several types of cancers (116–118). Oxaliplatin, considered an ICD inducer chemotherapeutic agent, has been found to induce parthanatos in oral squamous cell carcinoma (OSCC) cells *in vitro* and *in vivo* (119). Cytarabine/AraC is known to induce apoptosis in several types of acute myeloid leukemia (AML) cell lines. However, a recent study has revealed that AraC also induces parthanatos in OCI-AML3 cells, suggesting that cell type or AraC sensitivity may alter the consequences of AraC treatment (120).

A recent study shows that mixed lineage kinase domain-like pseudokinase (MLKL) deficiency in hepatocellular carcinoma cells restricts ER Mg2+ release and mitochondrial Mg2+ uptake, leading to ER dysfunction, mitochondrial oxidative stress, and, ultimately, metabolic-stress-induced parthanatos (121). Therefore, MLKL deficiency in HCC cells suppresses orthotopic tumor growth, activates the anti-tumor immune response, and enhances the therapeutic effect of immune checkpoint blockade in syngeneic HCC tumor mouse models. The use of a PARP-1 inhibitor to block parthanatos could then restore tumor growth and immune evasion in MLKL-knockout HCC tumors. Since MLKL is a critical executioner of necroptosis, this report elucidates a new role for MLKL in negatively regulating parthanatos in HCC.

### Alkaliptosis

Alkaliptosis, a pH-dependent regulated cell death process triggered by the small molecular compound JTC801, has recently been identified as a novel approach for malignant tumor treatment, particularly in pancreatic cancer through the screening of new anti-cancer drugs (122, 123). Indeed, *in vivo* experiments in mice showed that JTC801 selectively targets pancreatic ductal adenocarcinoma (PDAC) cells without harming normal cells. Two major signaling pathways, the ATPase H+ transporting V0 subunit D1 (ATP6V0D1)-signal transducer and activator of transcription 3 (STAT3) pathway and the NF-κB pathway, are reported to contribute to the induction of alkaliptosis (122, 124, 125). Blocking other cell death modes such as apoptosis, necroptosis, and ferroptosis did not prevent JTC801-induced cell death. Mass spectrometry identified ATP6V0D1 as a JTC801 target (125). ATP6V0D1, a member of the vacuolar ATPase (V-ATPase) family, regulates the acidification of intracellular organelles. It forms a complex with STAT3 in lysosomes, leading to lysosomal acidification and cytosolic alkalinization, thus inducing alkaliptosis. However, inhibiting ATP6V0D1 fails to prevent JTC801-induced lysosomal acidification, indicating the potential involvement of a compensatory mechanism (126). Therefore, the precise mechanism remains unclear. JTC801 activates NF-κB-dependent carbonic anhydrase 9 (CA9) downregulation (127). CA9 regulates pH by catalyzing the reversible hydration of carbon dioxide to carbonic acid and is overexpressed in many types of solid cancers, including PDAC, where it promotes tumor growth by inhibiting intracellular alkalinization. JTC801-induced alkaliptosis is capable of activating NF-kb making it another potential means of inducing ICD. Most importantly, JTC801-induced alkaliptosis of cancer cells can lead to the release of HMGB1 into the cell culture supernatants. This release of HMGB1 from the nucleus involves nuclear DNA damage signaling and can be inhibited by the FA complementation group D2 (FANCD2)-dependent DNA repair pathways (128). It has been reported that extracellular HMGB1 binds to its receptor, advanced glycosylation end-product-specific receptor (AGER, also known as RAGE), in macrophages and activates the cGAS-STING pathway-mediated immunity (128). Another recent report revealed that acyl-CoA synthetase short-chain family member 2 (ACSS2), responsible for producing acetyl-CoA and leading to NF-κB acetylation, subsequently activates the NF-κB pathway and CA9 downregulation, promoting alkaliptosis in PDAC cells (129). Thus, alkaliptosis is potentially ICD-like, but it is not well-characterized thus far as a cancer immunotherapy and needs to be further examined in the future.

### Autophagy and autosis

Autophagy, a well-known programmed cell death mode, plays a paradoxical role in the anti-tumor response. Autophagy is a self-degradative type of cell death accompanied by large-scale autophagic vacuolization of the cytoplasm, resulting in a vacuolated appearance in response to stressors, such as nutrient deprivation (130). Autophagy also serves as an advanced system for the elimination of DAMPs and PAMPs, contributing to the maintenance of immune homeostasis (131–134). In this role, autophagy basically suppresses inflammatory cytokine production and inflammasome activation, reducing ICD-mediated immune activation. Autophagy also aids tumor cells in evading immune surveillance by inhibiting antitumor immune responses. For example, autophagy degrades the MHC-I complex, thereby preventing immune cell recognition in a pancreatic cancer mouse model (135, 136). In contrast, the autophagy inhibitor chloroquine (CQ) increases MHC-I complexes and results in enhanced antitumor immune responses (135). Overall, evidence suggests that autophagy is immunosuppressive and linked to cancer survival and proliferation. However, several studies have also revealed that autophagy also contributes to antitumor functions depending on its level of activation or strength (137, 138). High-level autophagy and not mild autophagy, is able to promote ICD in tumor cells by releasing DAMPs from dead cells, which can inflame the TME and activate anti-tumor immunity. During ICD, autophagosomes including tumor antigens and DAMPs are released into extracellular space and taken up by APCs (139). Mechanistically, during autophagy in response to ICD induction, it has been reported that ATP is secreted from the plasma membrane through lysosomes and autolysosomes (17). Most importantly, suppression of autophagy results in diminished release of ATP and DAMPs from dying tumor cells treated with anthracycline chemotherapeutic agents, indicating an essential role of autophagy in ICD (140, 141). Autophagy has also recently been demonstrated to have a role in necroptosis. Autophagy induction by obatoclax results in FADD/RIPK1/RIPK3 recruitment to the autophagosomal membranes by interaction with Atg5, suggesting that autophagy may promote necroptosis via the assembly of the necrosome on autophagosomes (142). Thus, high-level autophagy is capable of promoting cancer cell death and releasing DAMPs from deceased cells. This process can remodel the TME and activate anti-tumor immunity (138).

Recently, a novel form of autophagy gene-dependent, Na^+^,K^+^-ATPase-regulated, non-apoptotic cell death, termed ‘autosis’, which is induced by excessive autophagy, prolonged autophagy-inducing peptides (HIV1-Tat fused Beclin 1, Tat-BECN1) treatment, starvation, and hypoxia-ischemia, and characterized by the disappearance of the endoplasmic reticulum and focal swelling of the perinuclear space has been identified (143, 144). Autosis is associated with plasma membrane rupture, suggesting the release of intracellular components that can increase immunogenicity. Indeed, it has been reported that Tat-BECN1 treatment triggered autosis enhances immunogenicity *in vivo* (145).

It has also been recently reported that myxoma virus (MYXV)-infected tumor-specific T (T^MYXV^) cells expressing a chimeric antigen receptor (CAR), ‘CAR-T^MXYV^’, eradicate antigen-loss tumors by inducing tumor autosis (146). Mechanistically, cytotoxic T cell-derived IFNγ synergistically activates CAR-T^MXYV^ mediated autophagosome formation and executes cancer cell autosis. This method can induce both cytotoxicity and tumor antigen MYXV to antigen-deficient (cold) tumor bed in a B16 melanoma mouse model, resulting in a boost to adaptive immunity similar to ICD induction.

Depending on the cancer cell types and circumstances in the TME, autophagy can be both anti-cancer and pro-cancer, which should raise concerns when choosing autophagy modulators for clinical studies and use (147, 148). Therefore, autosis induction, instead of autophagy inhibition or induction, may be a more reasonable target for consideration as an anti-cancer treatment.

### Pyroptosis

The term “pyroptosis” was originally coined to indicate proinflammatory (from the Greek “pyro” referring to fire) programmed cell death (“ptosis”) during bacterial infection in macrophages. Now, it is known that pyroptosis also occurs in cancer cells and induces anti-tumor immunity (43, 149, 150). Pyroptosis is a programmed necrotic cell death mediated by pore-forming gasdermin (GSDM) proteins (151). Morphological characteristics of pyroptosis include cytoplasmic swelling, DNA fragmentation, and pore formation, resulting in the emission of intracellular contents and proinflammatory cytokines (e.g., IL-1β, IL-18, IL-33, and HMGB1). Pyroptosis is executed by inflammasome-dependent or -independent mechanisms (152). Inflammasomes, multi-protein oligomers, are divided into NOD-like receptors (NLRs) (e.g., NLRP1, NLRP3, and NLRC4, with NLRP3 being the most-studied inflammasome) and non-NLRs (e.g., AIM2; AIM2 is activated by cytosolic bacterial, viral, and host dsDNA) (153–156). The inflammasome is activated by PAMPs and DAMPs and triggers the activation of caspase-1, known as canonical inflammasome activation. Active caspase-1 cleaves gasdermin D (GSDMD) and further matures IL-1β and IL-18, leading to pyroptosis (157–159). LPS also activates the non-canonical inflammasome, targeting caspase-4/5/11 (human caspase-4/5 and mouse caspase-11), followed by cleavage of GSDMD (160–165). The cleaved N-terminal GSDMD acts as the “executioner”, forming pores in the cell membrane (166, 167). Gasdermin E (GSDME, also called DFNA5) was recently identified as another pyroptotic “executioner” and suppresses tumor growth by activating anti-tumor immunity (168). During chemotherapy, in GSDME expressing cells, GSDME can be cleaved and activated by caspase-3, converting cells from apoptotic to secondary necrotic/pyroptotic (169, 170). In the TME of GSDME expressing tumors (breast, colorectal and melanoma), the function and number of DCs, cytotoxic lymphocytes and NK cells are increased (168). Another study demonstrates that the combination of a BRAF inhibitor and MEK inhibitor activates caspase-3 GSDME-mediated pyroptosis in melanoma, leading to increased immune response and tumor suppression in melanoma-bearing mice (171). It has also been reported that the cyclin-dependent kinase (CDK) -1, -2 and -9 inhibitor, Dinaciclib, induces ICD and has confirmed anti-tumor effects in syngeneic MC38, CT26 colon and MB49 bladder cancer mouse models (172). Dinaciclib-mediated ICD has been further evaluated as a GSDME-mediated pyroptosis in several triple negative breast cancer cell lines and a 4T1 breast cancer mouse model (173). Notably, GSDME is silenced in multiple tumors through GSDME DNA methylation, and therefore, methyltransferase inhibitors (e.g., decitabine) can increase GSDME expression and inhibit tumor growth (174, 175). Indeed, decreased GSDME expression in oral squamous cell carcinoma (OSCC) is associated with poor prognosis (176).Thus, this evidence suggests that GSDME mediated pyroptosis plays an essential role in tumor suppression. In addition to GSDMD- or GSDME-mediated pyroptosis, other GSDM-mediated pyroptosis pathways have been reported. For example, granzymes (Gzms) secreted from CTL or NK cells can induce pyroptosis. GzmA cleaves GSDMB and triggers pyroptosis (177). GzmB cleaves both Caspase-3 and GSDME, amplifying GzmB-mediated pyroptosis (168, 178, 179). Since cancer pyroptosis results in immune cell activation and these activated cells secrete granzymes in the TME, induction of cancer pyroptosis may be one of the reasonable cancer immunotherapeutic strategies. Whether there are any other additional mechanisms of cancer pyroptosis will require further investigation.

### Atypical ISGs induced pyroptosis

In most instances, type I IFNs are crucial for subsequent immune response activation but are not mandatory for the induction of ICD itself. Notably, the release of type I IFNs serves as a biomarker for the completion of ICD (**Figure 1**) (37). However, recent evidence highlights that, beyond type I IFN production, the response of tumor cells to type I IFNs represents an essential pathway for eliciting effective antitumor responses following ICD induction. For instance, the induction of ICD by anthracycline-based chemotherapies and radiotherapy relies heavily on their capacity to activate IFN-dependent gene expression programs within tumor cells, thereby facilitating the generation of robust antitumor immune responses (180, 181). Regarding whether modulating the IFN response promotes ICD, it was recently shown that the IFN inducible Z-form nucleic acid binding protein 1 (ZBP1) can trigger ICD in *Ripk1*-/- cells (182). However, ZBP1 induction by IFN alone appears insufficient to induce ICD, implying the involvement of other unidentified factors. Moreover, although cGAS-STING or retinoic acid inducible gene I (RIG-I) pathway activation can induce ICD, the precise mechanisms beyond type I IFN production remain unclear (183). While numerous studies underscore the significance of the type I IFN response in ICD induction and its intrinsic ability to enhance antitumor immunity, the extent to which modulating or enhancing IFN responses directly promotes ICD remains insufficiently characterized. Consequently, it is hypothesized that genetic factors regulate IFN-mediated ICD, and targeting these factors may hold therapeutic promise, particularly considering the elevated levels of interferon production in the tumor microenvironment.

The production and signaling of type I IFNs are tightly regulated (184). Our recent discovery reveals that the depletion of ubiquitin-specific protease 18 (USP18), a major negative regulator of IFN signaling, selectively induces cancer cell ICD, specifically pyroptosis (185, 186). We identified that nuclear USP18 diminishes binding of IFN regulated transcription factors to their corresponding DNA motifs in cooperation with NF-κB. Consequently, the suppression of USP18 not only enhances the expression of canonical IFN-stimulated genes (ISGs) but also activates a set of atypical ISGs and NF-κB target genes that induce cancer pyroptosis. Importantly, partial loss of *Usp18* in mice does not disrupt normal hematopoiesis, and mice and humans heterozygous for Usp18 are healthy and normal (186–188). Our recent study further demonstrates that USP18 depletion in the myeloid lineage exerts an anti-cancer effect by reprogramming M2 macrophages to M1 macrophages in the tumor microenvironment (189). These findings suggest a promising therapeutic opportunity for targeting USP18 in clinical applications.

Additionally, we observed a clear translocation of HMGB1 from the nucleus to the cytoplasm in *Usp18* ^+/-^ and *Usp18* ^-/-^ MC38 colon tumors, representing an ICD associated event in solid tumors. Significantly, higher infiltration of CD8^+^ T cells was noted in these tumors, suggesting that the depletion of *Usp18* in solid tumors can induce ICD and enhance the immune response. We also observed higher numbers of CD8^+^ T and activated CD8^+^ T cells in host splenocytes of recipients of *Usp18* ^+/Δ^ AML cells (186). Finally, a significant reduction in tumor development was observed in the group of mice vaccinated with IFN-treated *Usp18* ^+/-^ cells, as assessed by the gold standard vaccination assay in B16F10 and MC38 tumor mouse models. Importantly, this vaccine effect is attributed to the enhancement of tumor-infiltrating activated CD8^+^ T cells, a crucial component for the ICD-induced vaccine response. Thus, ICD induced by targeting *Usp18* occurs in certain solid cancers (186).

Among the atypical ISGs in IFN treated *USP18* depleted cancer cells, we identified PLK2 as one mediator of the observed ICD (186). Enhanced levels of *PLK2* correlated with high levels of caspase-3 and GSDME cleavage, a hallmark of pyroptosis, in IFN treated *USP18^-/-^* cancer cells. Importantly, we demonstrated that the ectopic expression of PLK2, independent of its kinase activity, in several types of cancer cells promoted the caspase-3 processing, GSDME cleavage, DAMPs release, and cell death, albeit without IL-1β secretion. Since the NLRP3 inflammasome can be formed and activated by DAMPs, PLK2-induced DAMP release could also be crucial for GSDMD pathway activation in the USP18-depleted tumor environment *in vivo*. Notably, the suppression of PLK2 kinase activity enhances the protein level of PLK2. Consequently, both a USP18 inhibitor and a PLK2 kinase inhibitor can induce cancer pyroptosis and hold significant potential as cancer therapeutic agents (186).

### Lysosomal cell death

Lysosomal cell death (LCD), is a form of RCD mediated by lysosomal damage triggered lysosomal membrane permeabilization (LMP). Christian de Duve first identified LCD in 1983, and the term “lysosomal cell death” was later coined in 2000 (190, 191). Lysosomal damage can be triggered by various agents such as chloroquine, lipid metabolites, ROS, and certain anti-cancer drugs (e.g., sorafenib). Growing evidence suggests that mild lysosomal damage results in cell apoptosis, while extensive damage can induce ICD. This highlights LCD as a rational target for promising cancer immunotherapy.

During LMP, the release of cathepsins and ROS plays a crucial role in ICD. For example, cathepsin D mediated necroptosis, cathepsin B- or G- mediated pyroptosis and ROS-mediated ferroptosis have been reported (192–197). A recent study demonstrated that the lysosomal inhibitor DC661 induces lysosomal lipid peroxidation followed by LMP and ICD, involving necroptosis, ferroptosis, and pyroptosis (198). Interestingly, these forms of cell death cannot be rescued by either ICD inhibitors or cathepsin inhibitors but can be reversed by the antioxidant N-acetylcysteine (NAC), highlighting the uniqueness of DC661-induced specific LCD. Importantly, mice vaccinated with DC661-treated cells exhibited secondary tumor rejection, a critical indication that DC661-mediated tumor cell death is immunogenic in nature.

## Discussion

Altogether, these findings suggest that appropriate induction of ICD can lead to successful control of multiple neoplasms through (immuno)therapy. ICD has garnered significant attention in cancer research, as it possesses the capability to enhance the body’s immune response to recognize and eliminate cancer cells.

In this review, we provide a summary of both previous and recent studies related to cancer ICD. Notably, numerous novel cell death modalities have been discovered within the last 20 years, cultivating a deeper understanding of which cell death modes exhibit ICD-like characteristics (e.g., paraptosis is now recognized as ICD). Moreover, our study revealed that hyper type I IFN response-mediated atypical ISGs selectively induce cancer ICD. It is increasingly evident that a single cell death mode can transition to different types of cell death, including ICD, under specific conditions. These immunostimulatory properties of ICD render ICD-inducing agents attractive candidates for cancer immune-monotherapy, with initial results from *in vitro* experiments and preclinical models suggesting a promising, viable path forward. However, numerous unknown factors still impede progress in ICD-mediated cancer immunotherapy, particularly concerning the development of reagents for tumor-specific ICD induction in clinical settings, strategies for reliably inducing ICD across different cancer types without inducing toxicity and resistance, and understanding the mechanisms by which various ICD modes enable protective anti-tumor immunity.

Currently, only a limited number of cytotoxic agents and methodologies (e.g., anthracyclines, oxaliplatin, radiation therapy, oncolytic viruses) have demonstrated the ability to induce ICD, with approved clinical use restricted to certain cancer types due to the genetic diversity, tissue origin, and local microenvironment of the tumor (35). Therefore, researchers are also actively exploring novel, clinically viable combinatorial strategies to enhance therapeutic potential by combining ICD with other approved regimens, including ICB (172, 199). Indeed, several combinations of ICD inducers are currently being investigated in clinical trials for specific types of cancer (8, 200, 201). Moreover, recent studies reveal that certain compounds/agents (or their combinations) have ICD-inducing abilities with different cell death modalities (202, 203). It is also noteworthy that strategies for drug delivery, including nanoparticles-mediated ICD induction, are under development (97, 204–206). However, the majority of agents/methodologies for ICD induction have only been tested through *in vitro* experiments and preclinical animal models. Further investigations, including clinical studies involving newly identified agents/methodologies with a focus on ICD, will provide more valuable evidence for the treatment of cancer. We anticipate that the evolution of ICD induction from current and future studies will increasingly take center stage in modern cancer control.

## Author contributions

K-iA: Conceptualization, Writing – original draft. SM: Writing – review & editing. ML: Writing – review & editing. D-EZ: Funding acquisition, Supervision, Writing – review & editing.

## Glossary

**Table:**

ICB	immune checkpoint blockade
ICD	immunogenic cell death
RCD	regulated cell death
PLK2	polo like kinase 2
TME	tumor microenvironment
TGF-β	transforming growth factor-β
DAMPs	damage-associated molecular patterns
cDAMPs	constitutive DAMPs
iDAMPs	inducible DAMPs
CXCL10	C-X-C motif chemokine ligand 10
PAMPs	pathogen-associated molecular patterns
HMGB1	nuclear protein high mobility group box 1
ATP	adenosine triphosphate
APC	antigen-presenting cell
DC	dendritic cell
TLR	toll-like receptor
RAGE	receptors for advanced glycation end-products
IL-1 b	interleukin 1b
CTL	cytotoxic T lymphocyte
CRT	calreticulin
MHC	major histocompatibility complex
AIM2	absent in melanoma 2
NK	natural killer
LCD	lysosomal cell death
ACD	accidental cell death
CrmA	cytokine response modifier A
TNFα	tumor necrosis factor-α
TRAIL	TNF-related apoptosis-inducing ligand
FADD	fas-associated via death domain
PRR	pattern recognition receptor
DAI	DNA-dependent activator of interferon-regulatory factors
cGAS	cyclic GMP-AMP synthase
RIPK	receptor-interacting protein kinase
MLKL	mixed lineage kinase domain-like pseudokinase
NNSCC	head and neck squamous cell carcinoma
HCC	hepatocellular carcinoma
ROS	reactive oxygen species
GSH	glutathione
SLC7A11	solute carrier family 7 member 11
GPX4	glutathione peroxidase 4
RCC	renal cell carcinoma
NADPH	nicotinamide adenine dinucleotide phosphate
Cu	Copper
TCA	tricarboxylic acid
DLAT	dihydrolipoamide S-acetyltransferase
FDX1	ferredoxin 1
LIAS	lipoyl synthase
TTM	tetrathiomolybdate
ER	endoplasmic reticulum
NSCLC	non-small-cell lung cancer
IGFIR	insulin-like growth factor I receptor
PERK	pancreatic ER kinase (PKR)-like ER kinase
eIF2α	eukaryotic translation initiation factor 2α
ATF4	activating transcription factor 4
IDO	indoleamine 2,3-dioxygenase
PARP1	poly (ADP-ribose) polymerase 1
AIF	apoptosis-inducing factor
MIF	macrophage migration inhibitory factor
OSCC	oral squamous cell carcinoma
AML	acute myeloid leukemia
PDAC	pancreatic ductal adenocarcinoma
ATP6V0D1	ATPase H+ transporting V0 subunit D1
STAT3	signal transducer and activator of transcription 3
V-ATPase	vacuolar ATPaseCA9: carbonic anhydrase 9
FANCD2	FA complementation group D2
ACSS2	acyl-CoA synthetase short-chain family member 2
CQ	chloroquine
CAR	chimeric antigen receptor
GSDM	gasdermin
NLR	NOD-like receptor
CDK	cyclin-dependent kinase
OSCC	oral squamous cell carcinoma
Gzm	granzyme
ZBP1	Z-form nucleic acid binding protein 1
RIG-I	retinoic acid inducible gene I
USP18	ubiquitin-specific protease 18
ISG	IFN-stimulated gene
LMP	lysosomal membrane permeabilization
NAC	N-acetylcysteine

## References

[1] Sharma P Allison JP. The future of immune checkpoint therapy. Science. (2015) 348:56–61. doi: 10.1126/science.aaa8172 25838373

[2] Shiravand Y Khodadadi F Kashani SMA Hosseini-Fard SR Hosseini S Sadeghirad H. Immune checkpoint inhibitors in cancer therapy. Curr Oncol. (2022) 29:3044–60. doi: 10.3390/curroncol29050247 PMC9139602 35621637

[3] Havel JJ Chowell D Chan TA. The evolving landscape of biomarkers for checkpoint inhibitor immunotherapy. Nat Rev Cancer. (2019) 19:133–50. doi: 10.1038/s41568-019-0116-x PMC6705396 30755690

[4] Waldman AD Fritz JM Lenardo MJ. A guide to cancer immunotherapy: from T cell basic science to clinical practice. Nat Rev Immunol. (2020) 20:651–68. doi: 10.1038/s41577-020-0306-5 PMC7238960 32433532

[5] Ansell SM Lesokhin AM Borrello I Halwani A Scott EC Gutierrez M. PD-1 blockade with nivolumab in relapsed or refractory Hodgkin’s lymphoma. N Engl J Med. (2015) 372:311–9. doi: 10.1056/NEJMoa1411087 PMC4348009 25482239

[6] Garon EB Rizvi NA Hui R Leighl N Balmanoukian AS Eder JP. Pembrolizumab for the treatment of non-small-cell lung cancer. N Engl J Med. (2015) 372:2018–28. doi: 10.1056/NEJMoa1501824 25891174

[7] Paucek RD Baltimore D Li G. The cellular immunotherapy revolution: arming the immune system for precision therapy. Trends Immunol. (2019) 40:292–309. doi: 10.1016/j.it.2019.02.002 30871979

[8] Choi M Shin J Lee CE Chung JY Kim M Yan X. Immunogenic cell death in cancer immunotherapy. BMB Rep. (2023) 56:275–86. doi: 10.5483/BMBRep.2023-0024 PMC10230015 37081756

[9] Palanivelu L Liu CH Lin LT. Immunogenic cell death: The cornerstone of oncolytic viro-immunotherapy. Front Immunol. (2022) 13:1038226. doi: 10.3389/fimmu.2022.1038226 36755812 PMC9899992

[10] Galluzzi L Buque A Kepp O Zitvogel L Kroemer G. Immunogenic cell death in cancer and infectious disease. Nat Rev Immunol. (2017) 17:97–111. doi: 10.1038/nri.2016.107 27748397

[11] Galon J Bruni D. Approaches to treat immune hot, altered and cold tumours with combination immunotherapies. Nat Rev Drug Discovery. (2019) 18:197–218. doi: 10.1038/s41573-018-0007-y 30610226

[12] Galluzzi L Humeau J Buque A Zitvogel L Kroemer G. Immunostimulation with chemotherapy in the era of immune checkpoint inhibitors. Nat Rev Clin Oncol. (2020) 17:725–41. doi: 10.1038/s41571-020-0413-z 32760014

[13] Kepp O Senovilla L Vitale I Vacchelli E Adjemian S Agostinis P. Consensus guidelines for the detection of immunogenic cell death. Oncoimmunology. (2014) 3:e955691. doi: 10.4161/21624011.2014.955691 25941621 PMC4292729

[14] Kroemer G Galluzzi L Kepp O Zitvogel L. Immunogenic cell death in cancer therapy. Annu Rev Immunol. (2013) 31:51–72. doi: 10.1146/annurev-immunol-032712-100008 23157435

[15] Casares N Pequignot MO Tesniere A Ghiringhelli F Roux S Chaput N. Caspase-dependent immunogenicity of doxorubicin-induced tumor cell death. J Exp Med. (2005) 202:1691–701. doi: 10.1084/jem.20050915 PMC2212968 16365148

[16] Tesniere A Panaretakis T Kepp O Apetoh L Ghiringhelli F Zitvogel L. Molecular characteristics of immunogenic cancer cell death. Cell Death Differ. (2008) 15:3–12. doi: 10.1038/sj.cdd.4402269 18007663

[17] Martins I Wang Y Michaud M Ma Y Sukkurwala AQ Shen S. Molecular mechanisms of ATP secretion during immunogenic cell death. Cell Death Differ. (2014) 21:79–91. doi: 10.1038/cdd.2013.75 23852373 PMC3857631

[18] Fucikova J Kepp O Kasikova L Petroni G Yamazaki T Liu P. Detection of immunogenic cell death and its relevance for cancer therapy. Cell Death Dis. (2020) 11:1013. doi: 10.1038/s41419-020-03221-2 33243969 PMC7691519

[19] Obeid M Panaretakis T Tesniere A Joza N Tufi R Apetoh L. Leveraging the immune system during chemotherapy: moving calreticulin to the cell surface converts apoptotic death from “silent” to immunogenic. Cancer Res. (2007) 67:7941–4. doi: 10.1158/0008-5472.CAN-07-1622 17804698

[20] Obeid M Tesniere A Panaretakis T Tufi R Joza N van Endert P. Ecto-calreticulin in immunogenic chemotherapy. Immunol Rev. (2007) 220:22–34. doi: 10.1111/j.1600-065X.2007.00567.x 17979837

[21] Obeid M Tesniere A Ghiringhelli F Fimia GM Apetoh L Perfettini JL. Calreticulin exposure dictates the immunogenicity of cancer cell death. Nat Med. (2007) 13:54–61. doi: 10.1038/nm1523 17187072

[22] Garg AD Krysko DV Verfaillie T Kaczmarek A Ferreira GB Marysael T. A novel pathway combining calreticulin exposure and ATP secretion in immunogenic cancer cell death. EMBO J. (2012) 31:1062–79. doi: 10.1038/emboj.2011.497 PMC3298003 22252128

[23] Stoll G Iribarren K Michels J Leary A Zitvogel L Cremer I. Calreticulin expression: Interaction with the immune infiltrate and impact on survival in patients with ovarian and non-small cell lung cancer. Oncoimmunology. (2016) 5:e1177692. doi: 10.1080/2162402X.2016.1177692 27622029 PMC5006900

[24] Fucikova J Kralikova P Fialova A Brtnicky T Rob L Bartunkova J. Human tumor cells killed by anthracyclines induce a tumor-specific immune response. Cancer Res. (2011) 71:4821–33. doi: 10.1158/0008-5472.CAN-11-0950 21602432

[25] Asadzadeh Z Safarzadeh E Safaei S Baradaran A Mohammadi A Hajiasgharzadeh K. Current approaches for combination therapy of cancer: the role of immunogenic cell death. Cancers (Basel). (2020) 12. doi: 10.20944/preprints202003.0228.v1 PMC7226590 32340275

[26] Murshid A Gong J Calderwood SK. The role of heat shock proteins in antigen cross presentation. Front Immunol. (2012) 3:63. doi: 10.3389/fimmu.2012.00063 22566944 PMC3342350

[27] Krysko DV Garg AD Kaczmarek A Krysko O Agostinis P Vandenabeele P. Immunogenic cell death and DAMPs in cancer therapy. Nat Rev Cancer. (2012) 12:860–75. doi: 10.1038/nrc3380 23151605

[28] Harding SM Benci JL Irianto J Discher DE Minn AJ Greenberg RA. Mitotic progression following DNA damage enables pattern recognition within micronuclei. Nature. (2017) 548:466–70. doi: 10.1038/nature23470 PMC5857357 28759889

[29] Yamazaki T Kirchmair A Sato A Buque A Rybstein M Petroni G. Mitochondrial DNA drives abscopal responses to radiation that are inhibited by autophagy. Nat Immunol. (2020) 21:1160–71. doi: 10.1038/s41590-020-0751-0 32747819

[30] Garg AD Galluzzi L Apetoh L Baert T Birge RB Bravo-San Pedro JM. Molecular and translational classifications of DAMPs in immunogenic cell death. Front Immunol. (2015) 6:588. doi: 10.3389/fimmu.2015.00588 26635802 PMC4653610

[31] Amarante-Mendes GP Adjemian S Branco LM Zanetti LC Weinlich R Bortoluci KR. Pattern recognition receptors and the host cell death molecular machinery. Front Immunol. (2018) 9:2379. doi: 10.3389/fimmu.2018.02379 30459758 PMC6232773

[32] Zitvogel L Kepp O Kroemer G. Decoding cell death signals in inflammation and immunity. Cell. (2010) 140:798–804. doi: 10.1016/j.cell.2010.02.015 20303871

[33] Schiavoni G Mattei F Gabriele L. Type I interferons as stimulators of DC-mediated cross-priming: impact on anti-tumor response. Front Immunol. (2013) 4:483. doi: 10.3389/fimmu.2013.00483 24400008 PMC3872318

[34] Parker BS Rautela J Hertzog PJ. Antitumour actions of interferons: implications for cancer therapy. Nat Rev Cancer. (2016) 16:131–44. doi: 10.1038/nrc.2016.14 26911188

[35] Tesniere A Schlemmer F Boige V Kepp O Martins I Ghiringhelli F. Immunogenic death of colon cancer cells treated with oxaliplatin. Oncogene. (2010) 29:482–91. doi: 10.1038/onc.2009.356 19881547

[36] Humeau J Levesque S Kroemer G Pol JG. Gold standard assessment of immunogenic cell death in oncological mouse models. Methods Mol Biol. (2019) 1884:297–315. doi: 10.1007/978-1-4939-8885-3_21 30465212

[37] Galluzzi L Vitale I Warren S Adjemian S Agostinis P Martinez AB. Consensus guidelines for the definition, detection and interpretation of immunogenic cell death. J Immunother Cancer. (2020) 8. doi: 10.1136/jitc-2019-000337 PMC7064135 32209603

[38] West NR Milne K Truong PT Macpherson N Nelson BH Watson PH. Tumor-infiltrating lymphocytes predict response to anthracycline-based chemotherapy in estrogen receptor-negative breast cancer. Breast Cancer Res. (2011) 13:R126. doi: 10.1186/bcr3072 22151962 PMC3326568

[39] Halama N Michel S Kloor M Zoernig I Benner A Spille A. Localization and density of immune cells in the invasive margin of human colorectal cancer liver metastases are prognostic for response to chemotherapy. Cancer Res. (2011) 71:5670–7. doi: 10.1158/0008-5472.CAN-11-0268 21846824

[40] Apetoh L Ghiringhelli F Tesniere A Obeid M Ortiz C Criollo A. Toll-like receptor 4-dependent contribution of the immune system to anticancer chemotherapy and radiotherapy. Nat Med. (2007) 13:1050–9. doi: 10.1038/nm1622 17704786

[41] Ghiringhelli F Apetoh L Tesniere A Aymeric L Ma Y Ortiz C. Activation of the NLRP3 inflammasome in dendritic cells induces IL-1beta-dependent adaptive immunity against tumors. Nat Med. (2009) 15:1170–8. doi: 10.1038/nm.2028 19767732

[42] Miao YD Quan W Dong X Gan J Ji CF Wang JT. A bibliometric analysis of ferroptosis, necroptosis, pyroptosis, and cuproptosis in cancer from 2012 to 2022. Cell Death Discovery. (2023) 9:129. doi: 10.1038/s41420-023-01421-1 37061535 PMC10105750

[43] Tang R Xu J Zhang B Liu J Liang C Hua J. Ferroptosis, necroptosis, and pyroptosis in anticancer immunity. J Hematol Oncol. (2020) 13:110. doi: 10.1186/s13045-020-00946-7 32778143 PMC7418434

[44] Degterev A Huang Z Boyce M Li Y Jagtap P Mizushima N. Chemical inhibitor of nonapoptotic cell death with therapeutic potential for ischemic brain injury. Nat Chem Biol. (2005) 1:112–9. doi: 10.1038/nchembio711 16408008

[45] Ray CA Pickup DJ. The mode of death of pig kidney cells infected with cowpox virus is governed by the expression of the crmA gene. Virology. (1996) 217:384–91. doi: 10.1006/viro.1996.0128 8599227

[46] Vercammen D Beyaert R Denecker G Goossens V Van Loo G Declercq W. Inhibition of caspases increases the sensitivity of L929 cells to necrosis mediated by tumor necrosis factor. J Exp Med. (1998) 187:1477–85. doi: 10.1084/jem.187.9.1477 PMC2212268 9565639

[47] Newton K Wickliffe KE Dugger DL Maltzman A Roose-Girma M Dohse M. Cleavage of RIPK1 by caspase-8 is crucial for limiting apoptosis and necroptosis. Nature. (2019) 574:428–31. doi: 10.1038/s41586-019-1548-x 31511692

[48] Fritsch M Gunther SD Schwarzer R Albert MC Schorn F Werthenbach JP. Caspase-8 is the molecular switch for apoptosis, necroptosis and pyroptosis. Nature. (2019) 575:683–7. doi: 10.1038/s41586-019-1770-6 31748744

[49] Upton JW Kaiser WJ Mocarski ES. DAI/ZBP1/DLM-1 complexes with RIP3 to mediate virus-induced programmed necrosis that is targeted by murine cytomegalovirus vIRA. Cell Host Microbe. (2019) 26:564. doi: 10.1016/j.chom.2019.09.004 31600504

[50] Jiao H Wachsmuth L Kumari S Schwarzer R Lin J Eren RO. Z-nucleic-acid sensing triggers ZBP1-dependent necroptosis and inflammation. Nature. (2020) 580:391–5. doi: 10.1038/s41586-020-2129-8 PMC7279955 32296175

[51] Decout A Katz JD Venkatraman S Ablasser A. The cGAS-STING pathway as a therapeutic target in inflammatory diseases. Nat Rev Immunol. (2021) 21:548–69. doi: 10.1038/s41577-021-00524-z PMC8029610 33833439

[52] Geng J Ito Y Shi L Amin P Chu J Ouchida AT. Regulation of RIPK1 activation by TAK1-mediated phosphorylation dictates apoptosis and necroptosis. Nat Commun. (2017) 8:359. doi: 10.1038/s41467-017-00406-w 28842570 PMC5572456

[53] Laurien L Nagata M Schunke H Delanghe T Wiederstein JL Kumari S. Autophosphorylation at serine 166 regulates RIP kinase 1-mediated cell death and inflammation. Nat Commun. (2020) 11:1747. doi: 10.1038/s41467-020-15466-8 32269263 PMC7142081

[54] Sun L Wang H Wang Z He S Chen S Liao D. Mixed lineage kinase domain-like protein mediates necrosis signaling downstream of RIP3 kinase. Cell. (2012) 148:213–27. doi: 10.1016/j.cell.2011.11.031 22265413

[55] Raju S Whalen DM Mengistu M Swanson C Quinn JG Taylor SS. Kinase domain dimerization drives RIPK3-dependent necroptosis. Sci Signal. (2018) 11. doi: 10.1126/scisignal.aar2188 PMC6310155 30131368

[56] Cai Z Jitkaew S Zhao J Chiang HC Choksi S Liu J. Plasma membrane translocation of trimerized MLKL protein is required for TNF-induced necroptosis. Nat Cell Biol. (2014) 16:55–65. doi: 10.1038/ncb2883 24316671 PMC8369836

[57] Van Hoecke L Van Lint S Roose K Van Parys A Vandenabeele P Grooten J. Treatment with mRNA coding for the necroptosis mediator MLKL induces antitumor immunity directed against neo-epitopes. Nat Commun. (2018) 9:3417. doi: 10.1038/s41467-018-05979-8 30143632 PMC6109072

[58] Snyder AG Hubbard NW Messmer MN Kofman SB Hagan CE Orozco SL. Intratumoral activation of the necroptotic pathway components RIPK1 and RIPK3 potentiates antitumor immunity. Sci Immunol. (2019) 4. doi: 10.1126/sciimmunol.aaw2004 PMC6831211 31227597

[59] Stoll G Ma Y Yang H Kepp O Zitvogel L Kroemer G. Pro-necrotic molecules impact local immunosurveillance in human breast cancer. Oncoimmunology. (2017) 6:e1299302. doi: 10.1080/2162402X.2017.1299302 28507808 PMC5414877

[60] Feng X Song Q Yu A Tang H Peng Z Wang X. Receptor-interacting protein kinase 3 is a predictor of survival and plays a tumor suppressive role in colorectal cancer. Neoplasma. (2015) 62:592–601. doi: 10.4149/neo_2015_071 25997957

[61] Hockendorf U Yabal M Herold T Munkhbaatar E Rott S Jilg S. RIPK3 restricts myeloid leukemogenesis by promoting cell death and differentiation of leukemia initiating cells. Cancer Cell. (2016) 30:75–91. doi: 10.1016/j.ccell.2016.06.002 27411587

[62] Geserick P Wang J Schilling R Horn S Harris PA Bertin J. Absence of RIPK3 predicts necroptosis resistance in Malignant melanoma. Cell Death Dis. (2015) 6:e1884. doi: 10.1038/cddis.2015.240 26355347 PMC4650439

[63] McCormick KD Ghosh A Trivedi S Wang L Coyne CB Ferris RL. Innate immune signaling through differential RIPK1 expression promote tumor progression in head and neck squamous cell carcinoma. Carcinogenesis. (2016) 37:522–9. doi: 10.1093/carcin/bgw032 PMC6086476 26992898

[64] Shi F Zhou M Shang L Du Q Li Y Xie L. EBV(LMP1)-induced metabolic reprogramming inhibits necroptosis through the hypermethylation of the RIP3 promoter. Theranostics. (2019) 9:2424–38. doi: 10.7150/thno.30941 PMC6525991 31131045

[65] Nicole L Sanavia T Cappellesso R Maffeis V Akiba J Kawahara A. Necroptosis-driving genes RIPK1, RIPK3 and MLKL-p are associated with intratumoral CD3(+) and CD8(+) T cell density and predict prognosis in hepatocellular carcinoma. J Immunother Cancer. (2022) 10. doi: 10.1136/jitc-2021-004031 PMC8915343 35264437

[66] Werthmoller N Frey B Wunderlich R Fietkau R Gaipl US. Modulation of radiochemoimmunotherapy-induced B16 melanoma cell death by the pan-caspase inhibitor zVAD-fmk induces anti-tumor immunity in a HMGB1-, nucleotide- and T-cell-dependent manner. Cell Death Dis. (2015) 6:e1761. doi: 10.1038/cddis.2015.129 25973681 PMC4669707

[67] Dixon SJ Lemberg KM Lamprecht MR Skouta R Zaitsev EM Gleason CE. Ferroptosis: an iron-dependent form of nonapoptotic cell death. Cell. (2012) 149:1060–72. doi: 10.1016/j.cell.2012.03.042 PMC3367386 22632970

[68] Stockwell BR. Ferroptosis turns 10: Emerging mechanisms, physiological functions, and therapeutic applications. Cell. (2022) 185:2401–21. doi: 10.1016/j.cell.2022.06.003 PMC9273022 35803244

[69] Clement CC Nanaware PP Yamazaki T Negroni MP Ramesh K Morozova K. Pleiotropic consequences of metabolic stress for the major histocompatibility complex class II molecule antigen processing and presentation machinery. Immunity. (2021) 54:721–36 e10. doi: 10.1016/j.immuni.2021.02.019 33725478 PMC8046741

[70] Jiang X Stockwell BR Conrad M. Ferroptosis: mechanisms, biology and role in disease. Nat Rev Mol Cell Biol. (2021) 22:266–82. doi: 10.1038/s41580-020-00324-8 PMC8142022 33495651

[71] Roh JL Kim EH Jang HJ Park JY Shin D. Induction of ferroptotic cell death for overcoming cisplatin resistance of head and neck cancer. Cancer Lett. (2016) 381:96–103. doi: 10.1016/j.canlet.2016.07.035 27477897

[72] Lei G Zhang Y Koppula P Liu X Zhang J Lin SH. The role of ferroptosis in ionizing radiation-induced cell death and tumor suppression. Cell Res. (2020) 30:146–62. doi: 10.1038/s41422-019-0263-3 PMC7015061 31949285

[73] Galluzzi L Kepp O Kroemer G. Immunogenic cell death in radiation therapy. Oncoimmunology. (2013) 2:e26536. doi: 10.4161/onci.26536 24404424 PMC3881599

[74] Efimova I Catanzaro E van der Meeren L Turubanova VD Hammad H Mishchenko TA. Vaccination with early ferroptotic cancer cells induces efficient antitumor immunity. J Immunother Cancer. (2020) 8. doi: 10.1136/jitc-2020-001369 PMC7668384 33188036

[75] Du Y Guo Z. Recent progress in ferroptosis: inducers and inhibitors. Cell Death Discovery. (2022) 8:501. doi: 10.1038/s41420-022-01297-7 36581640 PMC9800531

[76] Lachaier E Louandre C Godin C Saidak Z Baert M Diouf M. Sorafenib induces ferroptosis in human cancer cell lines originating from different solid tumors. Anticancer Res. (2014) 34:6417–22. 25368241

[77] Li Q Chen K Zhang T Jiang D Chen L Jiang J. Understanding sorafenib-induced ferroptosis and resistance mechanisms: Implications for cancer therapy. Eur J Pharmacol. (2023) 955:175913. doi: 10.1016/j.ejphar.2023.175913 37460053

[78] Guo L Hu C Yao M Han G. Mechanism of sorafenib resistance associated with ferroptosis in HCC. Front Pharmacol. (2023) 14:1207496. doi: 10.3389/fphar.2023.1207496 37351514 PMC10282186

[79] Liu X Nie L Zhang Y Yan Y Wang C Colic M. Actin cytoskeleton vulnerability to disulfide stress mediates disulfidptosis. Nat Cell Biol. (2023) 25:404–14. doi: 10.1038/s41556-023-01091-2 PMC10027392 36747082

[80] Liu X Zhuang L Gan B. Disulfidptosis: disulfide stress-induced cell death. Trends Cell Biol. (2023) 34:327–37. doi: 10.1016/j.tcb.2023.07.009 37574347

[81] Liu X Olszewski K Zhang Y Lim EW Shi J Zhang X. Cystine transporter regulation of pentose phosphate pathway dependency and disulfide stress exposes a targetable metabolic vulnerability in cancer. Nat Cell Biol. (2020) 22:476–86. doi: 10.1038/s41556-020-0496-x PMC7194135 32231310

[82] Joly JH Delfarah A Phung PS Parrish S Graham NA. A synthetic lethal drug combination mimics glucose deprivation-induced cancer cell death in the presence of glucose. J Biol Chem. (2020) 295:1350–65. doi: 10.1016/S0021-9258(17)49891-7 PMC6996897 31914417

[83] Shin CS Mishra P Watrous JD Carelli V D’Aurelio M Jain M. The glutamate/cystine xCT antiporter antagonizes glutamine metabolism and reduces nutrient flexibility. Nat Commun. (2017) 8:15074. doi: 10.1038/ncomms15074 28429737 PMC5413954

[84] Qi C Ma J Sun J Wu X Ding J. The role of molecular subtypes and immune infiltration characteristics based on disulfidptosis-associated genes in lung adenocarcinoma. Aging (Albany NY). (2023) 15:5075–95. doi: 10.18632/aging.204782 PMC10292876 37315289

[85] Li XM Liu SP Li Y Cai XM Zhang SB Xie ZF. Identification of disulfidptosis-related genes with immune infiltration in hepatocellular carcinoma. Heliyon. (2023) 9:e18436. doi: 10.1016/j.heliyon.2023.e18436 37520990 PMC10382636

[86] Chen X Liang Q Zhou Y. Construction of a novel disulfidptosis-related signature for improving outcomes in hepatocellular carcinoma: Observational study. Med (Baltimore). (2023) 102:e35423. doi: 10.1097/MD.0000000000035423 PMC10553138 37800779

[87] Zhao D Meng Y Dian Y Zhou Q Sun Y Le J. Molecular landmarks of tumor disulfidptosis across cancer types to promote disulfidptosis-target therapy. Redox Biol. (2023) 68:102966. doi: 10.1016/j.redox.2023.102966 38035663 PMC10698012

[88] Li Y Song Z Han Q Zhao H Pan Z Lei Z. Targeted inhibition of STAT3 induces immunogenic cell death of hepatocellular carcinoma cells via glycolysis. Mol Oncol. (2022) 16:2861–80. doi: 10.1002/1878-0261.13263 PMC9348600 35665592

[89] Tsvetkov P Coy S Petrova B Dreishpoon M Verma A Abdusamad M. Copper induces cell death by targeting lipoylated TCA cycle proteins. Science. (2022) 375:1254–61. doi: 10.1126/science.abf0529 PMC9273333 35298263

[90] Tang D Chen X Kroemer G. Cuproptosis: a copper-triggered modality of mitochondrial cell death. Cell Res. (2022) 32:417–8. doi: 10.1038/s41422-022-00653-7 PMC9061796 35354936

[91] Tsvetkov P Detappe A Cai K Keys HR Brune Z Ying W. Mitochondrial metabolism promotes adaptation to proteotoxic stress. Nat Chem Biol. (2019) 15:681–9. doi: 10.1038/s41589-019-0291-9 PMC8183600 31133756

[92] Brewer GJ Askari F Dick RB Sitterly J Fink JK Carlson M. Treatment of Wilson’s disease with tetrathiomolybdate: V. Control of free copper by tetrathiomolybdate and a comparison with trientine. Transl Res. (2009) 154:70–7. doi: 10.1016/j.trsl.2009.05.002 19595438

[93] Chen L Min J Wang F. Copper homeostasis and cuproptosis in health and disease. Signal Transduct Target Ther. (2022) 7:378. doi: 10.1038/s41392-022-01229-y 36414625 PMC9681860

[94] Kaur P Johnson A Northcote-Smith J Lu C Suntharalingam K. Immunogenic cell death of breast cancer stem cells induced by an endoplasmic reticulum-targeting copper(II) complex. Chembiochem. (2020) 21:3618–24. doi: 10.1002/cbic.202000553 PMC7757018 32776422

[95] Liu J Liu Y Wang Y Kang R Tang D. HMGB1 is a mediator of cuproptosis-related sterile inflammation. Front Cell Dev Biol. (2022) 10:996307. doi: 10.3389/fcell.2022.996307 36211458 PMC9534480

[96] Nagai M Vo NH Shin Ogawa L Chimmanamada D Inoue T Chu J. The oncology drug elesclomol selectively transports copper to the mitochondria to induce oxidative stress in cancer cells. Free Radic Biol Med. (2012) 52:2142–50. doi: 10.1016/j.freeradbiomed.2012.03.017 22542443

[97] Xu Y Liu SY Zeng L Ma H Zhang Y Yang H. An enzyme-engineered nonporous copper(I) coordination polymer nanoplatform for cuproptosis-based synergistic cancer therapy. Adv Mater. (2022) 34:e2204733. doi: 10.1002/adma.202204733 36054475

[98] An P Wei LL Zhao S Sverdlov DY Vaid KA Miyamoto M. Hepatocyte mitochondria-derived danger signals directly activate hepatic stellate cells and drive progression of liver fibrosis. Nat Commun. (2020) 11:2362. doi: 10.1038/s41467-020-16092-0 32398673 PMC7217909

[99] Davis CI Gu X Kiefer RM Ralle M Gade TP Brady DC. Altered copper homeostasis underlies sensitivity of hepatocellular carcinoma to copper chelation. Metallomics. (2020) 12:1995–2008. doi: 10.1039/d0mt00156b 33146201 PMC8315290

[100] Voli F Valli E Lerra L Kimpton K Saletta F Giorgi FM. Intratumoral copper modulates PD-L1 expression and influences tumor immune evasion. Cancer Res. (2020) 80:4129–44. doi: 10.1158/0008-5472.CAN-20-0471 32816860

[101] Xie Y Zhang W Sun J Sun L Meng F Yu H. A novel cuproptosis-related immune checkpoint gene signature identification and experimental validation in hepatocellular carcinoma. Sci Rep. (2022) 12:18514. doi: 10.1038/s41598-022-22962-y 36323801 PMC9630496

[102] Zhang Q Ma L Zhou H Zhou Y Liu S Li Q. A prognostic signature of cuproptosis and TCA-related genes for hepatocellular carcinoma. Front Oncol. (2022) 12:1040736. doi: 10.3389/fonc.2022.1040736 36324575 PMC9619237

[103] Cong T Luo Y Liu Y Yang C Yang H Li Y. Cuproptosis-related immune checkpoint gene signature: Prediction of prognosis and immune response for hepatocellular carcinoma. Front Genet. (2022) 13:1000997. doi: 10.3389/fgene.2022.1000997 36276933 PMC9579294

[104] Sperandio S de Belle I Bredesen DE. An alternative, nonapoptotic form of programmed cell death. Proc Natl Acad Sci U S A. (2000) 97:14376–81. doi: 10.1073/pnas.97.26.14376 PMC18926 11121041

[105] Li GN Zhao XJ Wang Z Luo MS Shi SN Yan DM. Elaiophylin triggers paraptosis and preferentially kills ovarian cancer drug-resistant cells by inducing MAPK hyperactivation. Signal Transduct Target Ther. (2022) 7:317. doi: 10.1038/s41392-022-01131-7 36097006 PMC9468165

[106] Liu S Tian Y Liu C Gui Z Yu T Zhang L. TNFRSF19 promotes endoplasmic reticulum stress-induced paraptosis via the activation of the MAPK pathway in triple-negative breast cancer cells. Cancer Gene Ther. (2023) 31:217–27. doi: 10.1038/s41417-023-00696-x 37990061

[107] Mandula JK Chang S Mohamed E Jimenez R Sierra-Mondragon RA Chang DC. Ablation of the endoplasmic reticulum stress kinase PERK induces paraptosis and type I interferon to promote anti-tumor T cell responses. Cancer Cell. (2022) 40:1145–60 e9. doi: 10.1016/j.ccell.2022.08.016 36150390 PMC9561067

[108] Xue J Li R Zhao X Ma C Lv X Liu L. Morusin induces paraptosis-like cell death through mitochondrial calcium overload and dysfunction in epithelial ovarian cancer. Chem Biol Interact. (2018) 283:59–74. doi: 10.1016/j.cbi.2018.02.003 29421517

[109] Pang HF Li XX Zhao YH Kang JK Li JY Tian W. Confirming whether novel rhein derivative 4a induces paraptosis-like cell death by endoplasmic reticulum stress in ovarian cancer cells. Eur J Pharmacol. (2020) 886:173526. doi: 10.1016/j.ejphar.2020.173526 32890460

[110] Munn DH Mellor AL. IDO in the tumor microenvironment: inflammation, counter-regulation, and tolerance. Trends Immunol. (2016) 37:193–207. doi: 10.1016/j.it.2016.01.002 26839260 PMC4916957

[111] Zheng RR Zhao LP Huang CY Cheng H Yang N Chen ZX. Paraptosis inducer to effectively trigger immunogenic cell death for metastatic tumor immunotherapy with IDO inhibition. ACS Nano. (2023) 17:9972–86. doi: 10.1021/acsnano.2c11964 37200049

[112] Andrabi SA Dawson TM Dawson VL. Mitochondrial and nuclear cross talk in cell death: parthanatos. Ann N Y Acad Sci. (2008) 1147:233–41. doi: 10.1196/annals.1427.014 PMC4454457 19076445

[113] David KK Andrabi SA Dawson TM Dawson VL. Parthanatos, a messenger of death. Front Biosci (Landmark Ed). (2009) 14:1116–28. doi: 10.2741/3297 PMC4450718 19273119

[114] Wang Y Dawson VL Dawson TM. Poly(ADP-ribose) signals to mitochondrial AIF: a key event in parthanatos. Exp Neurol. (2009) 218:193–202. doi: 10.1016/j.expneurol.2009.03.020 19332058 PMC2752872

[115] Wang Y An R Umanah GK Park H Nambiar K Eacker SM. A nuclease that mediates cell death induced by DNA damage and poly(ADP-ribose) polymerase-1. Science. (2016) 354. doi: 10.1126/science.aad6872 PMC5134926 27846469

[116] Zhao N Mao Y Han G Ju Q Zhou L Liu F. YM155, a survivin suppressant, triggers PARP-dependent cell death (parthanatos) and inhibits esophageal squamous-cell carcinoma xenografts in mice. Oncotarget. (2015) 6:18445–59. doi: 10.18632/oncotarget.v6i21 PMC4621902 26090615

[117] Boulos JC Omer EA Rigano D Formisano C Chatterjee M Leich E. Cynaropicrin disrupts tubulin and c-Myc-related signaling and induces parthanatos-type cell death in multiple myeloma. Acta Pharmacol Sin. (2023) 44:2265–81. doi: 10.1038/s41401-023-01117-3 PMC10618500 37344563

[118] Zhang Y Zhang C Li J Jiang M Guo S Yang G. Inhibition of AKT induces p53/SIRT6/PARP1-dependent parthanatos to suppress tumor growth. Cell Commun Signal. (2022) 20:93. doi: 10.1186/s12964-022-00897-1 35715817 PMC9205131

[119] Li D Kou Y Gao Y Liu S Yang P Hasegawa T. Oxaliplatin induces the PARP1-mediated parthanatos in oral squamous cell carcinoma by increasing production of ROS. Aging (Albany NY). (2021) 13:4242–57. doi: 10.18632/aging.v13i3 PMC7906208 33495407

[120] Maru B Messikommer A Huang L Seipel K Kovecses O Valk PJM. PARP-1 improves leukemia outcomes by inducing parthanatos during chemotherapy. Cell Rep Med. (2023) 4:101191. doi: 10.1016/j.xcrm.2023.101191 37683650 PMC10518631

[121] Jiang X Deng W Tao S Tang Z Chen Y Tian M. A RIPK3-independent role of MLKL in suppressing parthanatos promotes immune evasion in hepatocellular carcinoma. Cell Discovery. (2023) 9:7. doi: 10.1038/s41421-022-00504-0 36650126 PMC9845215

[122] Song X Zhu S Xie Y Liu J Sun L Zeng D. JTC801 induces pH-dependent death specifically in cancer cells and slows growth of tumors in mice. Gastroenterology. (2018) 154:1480–93. doi: 10.1053/j.gastro.2017.12.004 PMC5880694 29248440

[123] Liu J Kuang F Kang R Tang D. Alkaliptosis: a new weapon for cancer therapy. Cancer Gene Ther. (2020) 27:267–9. doi: 10.1038/s41417-019-0134-6 31467365

[124] Chen F Kang R Liu J Tang D. Mechanisms of alkaliptosis. Front Cell Dev Biol. (2023) 11:1213995. doi: 10.3389/fcell.2023.1213995 37601110 PMC10436304

[125] Chen F Zhu S Kang R Tang D Liu J. ATP6V0D1 promotes alkaliptosis by blocking STAT3-mediated lysosomal pH homeostasis. Cell Rep. (2023) 42:111911. doi: 10.1016/j.celrep.2022.111911 36640329

[126] Hu M Chen J Liu S Xu H. The acid gate in the lysosome. Autophagy. (2023) 19:1368–70. doi: 10.1080/15548627.2022.2125629 PMC10012942 36120744

[127] Swietach P Vaughan-Jones RD Harris AL. Regulation of tumor pH and the role of carbonic anhydrase 9. Cancer Metastasis Rev. (2007) 26:299–310. doi: 10.1007/s10555-007-9064-0 17415526

[128] Fang X Dai E Bai L Liu J Kang R Zhao Y. The HMGB1-AGER-STING1 pathway mediates the sterile inflammatory response to alkaliptosis. Biochem Biophys Res Commun. (2021) 560:165–71. doi: 10.1016/j.bbrc.2021.05.003 33992959

[129] Que D Kuang F Kang R Tang D Liu J. ACSS2-mediated NF-kappaB activation promotes alkaliptosis in human pancreatic cancer cells. Sci Rep. (2023) 13:1483. doi: 10.1038/s41598-023-28261-4 36707625 PMC9883393

[130] Kroemer G Levine B. Autophagic cell death: the story of a misnomer. Nat Rev Mol Cell Biol. (2008) 9:1004–10. doi: 10.1038/nrm2529 PMC2727358 18971948

[131] Nakahira K Haspel JA Rathinam VA Lee SJ Dolinay T Lam HC. Autophagy proteins regulate innate immune responses by inhibiting the release of mitochondrial DNA mediated by the NALP3 inflammasome. Nat Immunol. (2011) 12:222–30. doi: 10.1038/ni.1980 PMC3079381 21151103

[132] Jounai N Takeshita F Kobiyama K Sawano A Miyawaki A Xin KQ. The Atg5 Atg12 conjugate associates with innate antiviral immune responses. Proc Natl Acad Sci U S A. (2007) 104:14050–5. doi: 10.1073/pnas.0704014104 PMC1955809 17709747

[133] Saitoh T Fujita N Hayashi T Takahara K Satoh T Lee H. Atg9a controls dsDNA-driven dynamic translocation of STING and the innate immune response. Proc Natl Acad Sci U S A. (2009) 106:20842–6. doi: 10.1073/pnas.0911267106 PMC2791563 19926846

[134] Tal MC Sasai M Lee HK Yordy B Shadel GS Iwasaki A. Absence of autophagy results in reactive oxygen species-dependent amplification of RLR signaling. Proc Natl Acad Sci U S A. (2009) 106:2770–5. doi: 10.1073/pnas.0807694106 PMC2650341 19196953

[135] Yamamoto K Venida A Yano J Biancur DE Kakiuchi M Gupta S. Autophagy promotes immune evasion of pancreatic cancer by degrading MHC-I. Nature. (2020) 581:100–5. doi: 10.1038/s41586-020-2229-5 PMC7296553 32376951

[136] Van Kaer L Parekh VV Postoak JL Wu L. Role of autophagy in MHC class I-restricted antigen presentation. Mol Immunol. (2019) 113:2–5. doi: 10.1016/j.molimm.2017.10.021 29126597 PMC5940586

[137] Zhong Z Sanchez-Lopez E Karin M. Autophagy, inflammation, and immunity: A troika governing cancer and its treatment. Cell. (2016) 166:288–98. doi: 10.1016/j.cell.2016.05.051 PMC4947210 27419869

[138] Jin Z Sun X Wang Y Zhou C Yang H Zhou S. Regulation of autophagy fires up the cold tumor microenvironment to improve cancer immunotherapy. Front Immunol. (2022) 13:1018903. doi: 10.3389/fimmu.2022.1018903 36300110 PMC9589261

[139] Ma Y Galluzzi L Zitvogel L Kroemer G. Autophagy and cellular immune responses. Immunity. (2013) 39:211–27. doi: 10.1016/j.immuni.2013.07.017 23973220

[140] Michaud M Martins I Sukkurwala AQ Adjemian S Ma Y Pellegatti P. Autophagy-dependent anticancer immune responses induced by chemotherapeutic agents in mice. Science. (2011) 334:1573–7. doi: 10.1126/science.1208347 22174255

[141] Martins I Michaud M Sukkurwala AQ Adjemian S Ma Y Shen S. Premortem autophagy determines the immunogenicity of chemotherapy-induced cancer cell death. Autophagy. (2012) 8:413–5. doi: 10.4161/auto.19009 22361584

[142] Basit F Cristofanon S Fulda S. Obatoclax (GX15–070) triggers necroptosis by promoting the assembly of the necrosome on autophagosomal membranes. Cell Death Differ. (2013) 20:1161–73. doi: 10.1038/cdd.2013.45 PMC3741498 23744296

[143] Liu Y Shoji-Kawata S Sumpter RM Jr. Wei Y Ginet V Zhang L. Autosis is a Na+,K+-ATPase-regulated form of cell death triggered by autophagy-inducing peptides, starvation, and hypoxia-ischemia. Proc Natl Acad Sci U S A. (2013) 110:20364–71. doi: 10.1073/pnas.1319661110 PMC3870705 24277826

[144] Liu Y Levine B. Autosis and autophagic cell death: the dark side of autophagy. Cell Death Differ. (2015) 22:367–76. doi: 10.1038/cdd.2014.143 PMC4326571 25257169

[145] Shoji-Kawata S Sumpter R Leveno M Campbell GR Zou Z Kinch L. Identification of a candidate therapeutic autophagy-inducing peptide. Nature. (2013) 494:201–6. doi: 10.1038/nature11866 PMC3788641 23364696

[146] Zheng N Fang J Xue G Wang Z Li X Zhou M. Induction of tumor cell autosis by myxoma virus-infected CAR-T and TCR-T cells to overcome primary and acquired resistance. Cancer Cell. (2022) 40:973–85 e7. doi: 10.1016/j.ccell.2022.08.001 36027915 PMC9489043

[147] Galluzzi L Bravo-San Pedro JM Levine B Green DR Kroemer G. Pharmacological modulation of autophagy: therapeutic potential and persisting obstacles. Nat Rev Drug Discovery. (2017) 16:487–511. doi: 10.1038/nrd.2017.22 28529316 PMC5713640

[148] Mizushima N Levine B. Autophagy in human diseases. N Engl J Med. (2020) 383:1564–76. doi: 10.1056/NEJMra2022774 33053285

[149] Cookson BT Brennan MA. Pro-inflammatory programmed cell death. Trends Microbiol. (2001) 9:113–4. doi: 10.1016/S0966-842X(00)01936-3 11303500

[150] Wei X Xie F Zhou X Wu Y Yan H Liu T. Role of pyroptosis in inflammation and cancer. Cell Mol Immunol. (2022) 19:971–92. doi: 10.1038/s41423-022-00905-x PMC9376585 35970871

[151] Broz P Pelegrin P Shao F. The gasdermins, a protein family executing cell death and inflammation. Nat Rev Immunol. (2020) 20:143–57. doi: 10.1038/s41577-019-0228-2 31690840

[152] Broz P Dixit VM. Inflammasomes: mechanism of assembly, regulation and signalling. Nat Rev Immunol. (2016) 16:407–20. doi: 10.1038/nri.2016.58 27291964

[153] Swanson KV Deng M Ting JP. The NLRP3 inflammasome: molecular activation and regulation to therapeutics. Nat Rev Immunol. (2019) 19:477–89. doi: 10.1038/s41577-019-0165-0 PMC7807242 31036962

[154] Kelley N Jeltema D Duan Y He Y. The NLRP3 inflammasome: an overview of mechanisms of activation and regulation. Int J Mol Sci. (2019) 20. doi: 10.3390/ijms20133328 PMC6651423 31284572

[155] Fernandes-Alnemri T Yu JW Datta P Wu J Alnemri ES. AIM2 activates the inflammasome and cell death in response to cytoplasmic DNA. Nature. (2009) 458:509–13. doi: 10.1038/nature07710 PMC2862225 19158676

[156] Hornung V Ablasser A Charrel-Dennis M Bauernfeind F Horvath G Caffrey DR. AIM2 recognizes cytosolic dsDNA and forms a caspase-1-activating inflammasome with ASC. Nature. (2009) 458:514–8. doi: 10.1038/nature07725 PMC2726264 19158675

[157] Liu X Zhang Z Ruan J Pan Y Magupalli VG Wu H. Inflammasome-activated gasdermin D causes pyroptosis by forming membrane pores. Nature. (2016) 535:153–8. doi: 10.1038/nature18629 PMC5539988 27383986

[158] Shi J Zhao Y Wang K Shi X Wang Y Huang H. Cleavage of GSDMD by inflammatory caspases determines pyroptotic cell death. Nature. (2015) 526:660–5. doi: 10.1038/nature15514 26375003

[159] Xia S Zhang Z Magupalli VG Pablo JL Dong Y Vora SM. Gasdermin D pore structure reveals preferential release of mature interleukin-1. Nature. (2021) 593:607–11. doi: 10.1038/s41586-021-03478-3 PMC8588876 33883744

[160] Kayagaki N Warming S Lamkanfi M Vande Walle L Louie S Dong J. Non-canonical inflammasome activation targets caspase-11. Nature. (2011) 479:117–21. doi: 10.1038/nature10558 22002608

[161] Kayagaki N Stowe IB Lee BL O’Rourke K Anderson K Warming S. Caspase-11 cleaves gasdermin D for non-canonical inflammasome signalling. Nature. (2015) 526:666–71. doi: 10.1038/nature15541 26375259

[162] Kayagaki N Wong MT Stowe IB Ramani SR Gonzalez LC Akashi-Takamura S. Noncanonical inflammasome activation by intracellular LPS independent of TLR4. Science. (2013) 341:1246–9. doi: 10.1126/science.1240248 23887873

[163] Hagar JA Powell DA Aachoui Y Ernst RK Miao EA. Cytoplasmic LPS activates caspase-11: implications in TLR4-independent endotoxic shock. Science. (2013) 341:1250–3. doi: 10.1126/science.1240988 PMC3931427 24031018

[164] Shi J Zhao Y Wang Y Gao W Ding J Li P. Inflammatory caspases are innate immune receptors for intracellular LPS. Nature. (2014) 514:187–92. doi: 10.1038/nature13683 25119034

[165] Baker PJ Boucher D Bierschenk D Tebartz C Whitney PG D’Silva DB. NLRP3 inflammasome activation downstream of cytoplasmic LPS recognition by both caspase-4 and caspase-5. Eur J Immunol. (2015) 45:2918–26. doi: 10.1002/eji.201545655 26173988

[166] Gaidt MM Hornung V. Pore formation by GSDMD is the effector mechanism of pyroptosis. EMBO J. (2016) 35:2167–9. doi: 10.15252/embj.201695415 PMC5069554 27572465

[167] Sborgi L Ruhl S Mulvihill E Pipercevic J Heilig R Stahlberg H. GSDMD membrane pore formation constitutes the mechanism of pyroptotic cell death. EMBO J. (2016) 35:1766–78. doi: 10.15252/embj.201694696 PMC5010048 27418190

[168] Zhang Z Zhang Y Xia S Kong Q Li S Liu X. Gasdermin E suppresses tumour growth by activating anti-tumour immunity. Nature. (2020) 579:415–20. doi: 10.1038/s41586-020-2071-9 PMC7123794 32188940

[169] Wang Y Gao W Shi X Ding J Liu W He H. Chemotherapy drugs induce pyroptosis through caspase-3 cleavage of a gasdermin. Nature. (2017) 547:99–103. doi: 10.1038/nature22393 28459430

[170] Rogers C Fernandes-Alnemri T Mayes L Alnemri D Cingolani G Alnemri ES. Cleavage of DFNA5 by caspase-3 during apoptosis mediates progression to secondary necrotic/pyroptotic cell death. Nat Commun. (2017) 8:14128. doi: 10.1038/ncomms14128 28045099 PMC5216131

[171] Erkes DA Cai W Sanchez IM Purwin TJ Rogers C Field CO. Mutant BRAF and MEK inhibitors regulate the tumor immune microenvironment via pyroptosis. Cancer Discovery. (2020) 10:254–69. doi: 10.1158/2159-8290.CD-19-0672 PMC7007378 31796433

[172] Hossain DMS Javaid S Cai M Zhang C Sawant A Hinton M. Dinaciclib induces immunogenic cell death and enhances anti-PD1-mediated tumor suppression. J Clin Invest. (2018) 128:644–54. doi: 10.1172/JCI94586 PMC5785250 29337311

[173] Xu T Wang Z Liu J Wang G Zhou D Du Y. Cyclin-dependent kinase inhibitors function as potential immune regulators via inducing pyroptosis in triple negative breast cancer. Front Oncol. (2022) 12:820696. doi: 10.3389/fonc.2022.820696 35756622 PMC9213695

[174] Akino K Toyota M Suzuki H Imai T Maruyama R Kusano M. Identification of DFNA5 as a target of epigenetic inactivation in gastric cancer. Cancer Sci. (2007) 98:88–95. doi: 10.1111/j.1349-7006.2006.00351.x 17083569 PMC11158324

[175] Kim MS Chang X Yamashita K Nagpal JK Baek JH Wu G. Aberrant promoter methylation and tumor suppressive activity of the DFNA5 gene in colorectal carcinoma. Oncogene. (2008) 27:3624–34. doi: 10.1038/sj.onc.1211021 18223688

[176] Wang S Zhang MJ Wu ZZ Zhu SW Wan SC Zhang BX. GSDME is related to prognosis and response to chemotherapy in oral cancer. J Dent Res. (2022) 101:848–58. doi: 10.1177/00220345211073072 35148659

[177] Zhou Z He H Wang K Shi X Wang Y Su Y. Granzyme A from cytotoxic lymphocytes cleaves GSDMB to trigger pyroptosis in target cells. Science. (2020) 368. doi: 10.1126/science.aaz7548 32299851

[178] Darmon AJ Nicholson DW Bleackley RC. Activation of the apoptotic protease CPP32 by cytotoxic T-cell-derived granzyme B. Nature. (1995) 377:446–8. doi: 10.1038/377446a0 7566124

[179] Chowdhury D Lieberman J. Death by a thousand cuts: granzyme pathways of programmed cell death. Annu Rev Immunol. (2008) 26:389–420. doi: 10.1146/annurev.immunol.26.021607.090404 18304003 PMC2790083

[180] Burnette BC Liang H Lee Y Chlewicki L Khodarev NN Weichselbaum RR. The efficacy of radiotherapy relies upon induction of type i interferon-dependent innate and adaptive immunity. Cancer Res. (2011) 71:2488–96. doi: 10.1158/0008-5472.CAN-10-2820 PMC3070872 21300764

[181] Sistigu A Yamazaki T Vacchelli E Chaba K Enot DP Adam J. Cancer cell-autonomous contribution of type I interferon signaling to the efficacy of chemotherapy. Nat Med. (2014) 20:1301–9. doi: 10.1038/nm.3708 25344738

[182] Ingram JP Thapa RJ Fisher A Tummers B Zhang T Yin C. ZBP1/DAI drives RIPK3-mediated cell death induced by IFNs in the absence of RIPK1. J Immunol. (2019) 203:1348–55. doi: 10.4049/jimmunol.1900216 PMC6702065 31358656

[183] Duewell P Steger A Lohr H Bourhis H Hoelz H Kirchleitner SV. RIG-I-like helicases induce immunogenic cell death of pancreatic cancer cells and sensitize tumors toward killing by CD8(+) T cells. Cell Death Differ. (2014) 21:1825–37. doi: 10.1038/cdd.2014.96 PMC4227156 25012502

[184] Arimoto KI Miyauchi S Stoner SA Fan JB Zhang DE. Negative regulation of type I IFN signaling. J Leukoc Biol. (2018) 103:1099–116. doi: 10.1002/JLB.2MIR0817-342R 29357192

[185] Arimoto KI Lochte S Stoner SA Burkart C Zhang Y Miyauchi S. STAT2 is an essential adaptor in USP18-mediated suppression of type I interferon signaling. Nat Struct Mol Biol. (2017) 24:279–89. doi: 10.1038/nsmb.3378 PMC5365074 28165510

[186] Arimoto KI Miyauchi S Troutman TD Zhang Y Liu M Stoner SA. Expansion of interferon inducible gene pool via USP18 inhibition promotes cancer cell pyroptosis. Nat Commun. (2023) 14:251. doi: 10.1038/s41467-022-35348-5 36646704 PMC9842760

[187] Meuwissen ME Schot R Buta S Oudesluijs G Tinschert S Speer SD. Human USP18 deficiency underlies type 1 interferonopathy leading to severe pseudo-TORCH syndrome. J Exp Med. (2016) 213:1163–74. doi: 10.1084/jem.20151529 PMC4925017 27325888

[188] Alsohime F Martin-Fernandez M Temsah MH Alabdulhafid M Le Voyer T Alghamdi M. JAK inhibitor therapy in a child with inherited USP18 deficiency. N Engl J Med. (2020) 382:256–65. doi: 10.1056/NEJMoa1905633 PMC7155173 31940699

[189] Miyauchi S Arimoto KI Liu M Zhang Y Zhang DE. Reprogramming of tumor-associated macrophages via NEDD4-mediated CSF1R degradation by targeting USP18. Cell Rep. (2023) 42:113560. doi: 10.1016/j.celrep.2023.113560 38100351 PMC10822669

[190] de Duve C. Lysosomes revisited. Eur J Biochem. (1983) 137:391–7. doi: 10.1111/j.1432-1033.1983.tb07841.x 6319122

[191] Franko J Pomfy M Prosbova T. Apoptosis and cell death (mechanisms, pharmacology and promise for the future). Acta Med (Hradec Kralove). (2000) 43:63–8. doi: 10.14712/18059694.2019.115 10953379

[192] Bruchard M Mignot G Derangere V Chalmin F Chevriaux A Vegran F. Chemotherapy-triggered cathepsin B release in myeloid-derived suppressor cells activates the Nlrp3 inflammasome and promotes tumor growth. Nat Med. (2013) 19:57–64. doi: 10.1038/nm.2999 23202296

[193] Burgener SS Leborgne NGF Snipas SJ Salvesen GS Bird PI Benarafa C. Cathepsin G inhibition by serpinb1 and serpinb6 prevents programmed necrosis in neutrophils and monocytes and reduces GSDMD-driven inflammation. Cell Rep. (2019) 27:3646–56 e5. doi: 10.1016/j.celrep.2019.05.065 31216481 PMC7350907

[194] Liu S Li Y Choi HMC Sarkar C Koh EY Wu J. Lysosomal damage after spinal cord injury causes accumulation of RIPK1 and RIPK3 proteins and potentiation of necroptosis. Cell Death Dis. (2018) 9:476. doi: 10.1038/s41419-018-0469-1 29686269 PMC5913300

[195] Torii S Shintoku R Kubota C Yaegashi M Torii R Sasaki M. An essential role for functional lysosomes in ferroptosis of cancer cells. Biochem J. (2016) 473:769–77. doi: 10.1042/BJ20150658 26759376

[196] Gao H Bai Y Jia Y Zhao Y Kang R Tang D. Ferroptosis is a lysosomal cell death process. Biochem Biophys Res Commun. (2018) 503:1550–6. doi: 10.1016/j.bbrc.2018.07.078 30031610

[197] Zou J Kawai T Tsuchida T Kozaki T Tanaka H Shin KS. Poly IC triggers a cathepsin D- and IPS-1-dependent pathway to enhance cytokine production and mediate dendritic cell necroptosis. Immunity. (2013) 38:717–28. doi: 10.1016/j.immuni.2012.12.007 23601685

[198] Bhardwaj M Lee JJ Versace AM Harper SL Goldman AR Crissey MAS. Lysosomal lipid peroxidation regulates tumor immunity. J Clin Invest. (2023) 133. doi: 10.1172/JCI164596 PMC10104903 36795483

[199] Pfirschke C Engblom C Rickelt S Cortez-Retamozo V Garris C Pucci F. Immunogenic chemotherapy sensitizes tumors to checkpoint blockade therapy. Immunity. (2016) 44:343–54. doi: 10.1016/j.immuni.2015.11.024 PMC4758865 26872698

[200] Sprooten J Laureano RS Vanmeerbeek I Govaerts J Naulaerts S Borras DM. Trial watch: chemotherapy-induced immunogenic cell death in oncology. Oncoimmunology. (2023) 12:2219591. doi: 10.1080/2162402X.2023.2219591 37284695 PMC10240992

[201] Liu X Xie X Ren Y Shao Z Zhang N Li L. The role of necroptosis in disease and treatment. MedComm (2020). (2021) 2:730–55. doi: 10.1002/mco2.108 PMC8706757 34977874

[202] Wu Y Dong G Sheng C. Targeting necroptosis in anticancer therapy: mechanisms and modulators. Acta Pharm Sin B. (2020) 10:1601–18. doi: 10.1016/j.apsb.2020.01.007 PMC7563021 33088682

[203] Hanson S Dharan A P VS Pal S Nair BG Kar R. Paraptosis: a unique cell death mode for targeting cancer. Front Pharmacol. (2023) 14:1159409. doi: 10.3389/fphar.2023.1159409 37397502 PMC10308048

[204] Kim SE Zhang L Ma K Riegman M Chen F Ingold I. Ultrasmall nanoparticles induce ferroptosis in nutrient-deprived cancer cells and suppress tumour growth. Nat Nanotechnol. (2016) 11:977–85. doi: 10.1038/nnano.2016.164 PMC5108575 27668796

[205] Xu S Zheng H Ma R Wu D Pan Y Yin C. Vacancies on 2D transition metal dichalcogenides elicit ferroptotic cell death. Nat Commun. (2020) 11:3484. doi: 10.1038/s41467-020-17300-7 32661253 PMC7359333

[206] Zhang S Huang Y Pi S Chen H Ye F Wu C. Autophagy-amplifying nanoparticles evoke immunogenic cell death combined with anti-PD-1/PD-L1 for residual tumors immunotherapy after RFA. J Nanobiotechnology. (2023) 21:360. doi: 10.1186/s12951-023-02067-y 37789342 PMC10548684

